# Linking functions: an additional role for an intrinsically disordered linker domain in the transcriptional coactivator CBP

**DOI:** 10.1038/s41598-017-04611-x

**Published:** 2017-07-05

**Authors:** Sara Contreras-Martos, Alessandro Piai, Simone Kosol, Mihaly Varadi, Angela Bekesi, Pierre Lebrun, Alexander N. Volkov, Kris Gevaert, Roberta Pierattelli, Isabella C. Felli, Peter Tompa

**Affiliations:** 1 VIB-VUB Center for Structural Biology, Vlaams Instituut voor Biotechnologie (VIB), Brussel, Belgium; 2 Vrije Universiteit Brussel (VUB), Brussel, Belgium; 3 Magnetic Resonance Center, University of Florence, Florence, Italy; 4 Department of Chemistry “Ugo Schiff”, University of Florence, Florence, Italy; 5 Department of Chemistry, University of Warwick, Gibbet Hill, Coventry CV4 7AL United Kingdom; 6 Protein Data Bank in Europe, European Molecular Biology Laboratory, European Bioinformatics Institute (EMBL-EBI), Wellcome Genome Campus, Hinxton, Cambridge CB10 1SD UK; 7 VIB-UGent Center for Medical Biotechnology, Ghent, Belgium; 8 Institute of Enzymology, Research Centre for Natural Sciences of the Hungarian Academy of Sciences, Budapest, Hungary; 9 Department of Biochemistry, Ghent University, Ghent, Belgium

## Abstract

The multi-domain transcriptional coactivators CBP/p300 integrate a multitude of signaling inputs, interacting with more than 400 proteins via one or more of their globular domains. While CBP/p300 function is typically considered in terms of these structured domains, about half of the protein consists of intrinsically disordered regions (IDRs) of varying length. However, these IDRs have only been thought of as linkers that allow flexible spatial arrangement of the structured domains, but recent studies have shown that similar IDRs mediate specific and critical interactions in other proteins. To examine the roles of IDRs in CBP, we performed yeast-two-hybrid screenings of placenta and lung cancer cDNA libraries, which demonstrated that the long IDR linking the KIX domain and bromodomain of CBP (termed ID3) can potentially bind to several proteins. The RNA-binding Zinc-finger protein 106 (ZFP106) detected in both libraries was identified as a novel substrate for CBP-mediated acetylation. Nuclear magnetic resonance (NMR) spectroscopy combined with cross-linking experiments and competition-binding assays showed that the fully disordered isolated ID3 transiently interacts with an IDR of ZFP106 in a fashion that disorder of both regions is maintained. These findings demonstrate that beside the linking function, ID3 can also interact with acetylation substrates of CBP.

## Introduction

CREB-binding protein (CBP) and its homolog histone acetyltransferase p300 (p300) are multi-domain transcriptional regulators involved in diverse biological functions such as cell cycle, proliferation, differentiation, homeostasis, and apoptosis^1, 2^. Their function is mainly based on their interaction with a large variety of transcription factors and other regulatory proteins targeting their intrinsic histone acetyl transferase (HAT) activity on the chromatin and a broad range of partner proteins^3, 4^. Mutations affecting CBP/p300 genes have been implicated in Rubinstein-Taybi syndrome and several types of cancer, emphasizing their central role in orchestrating gene regulation^2, 3^.

**Figure 1:**
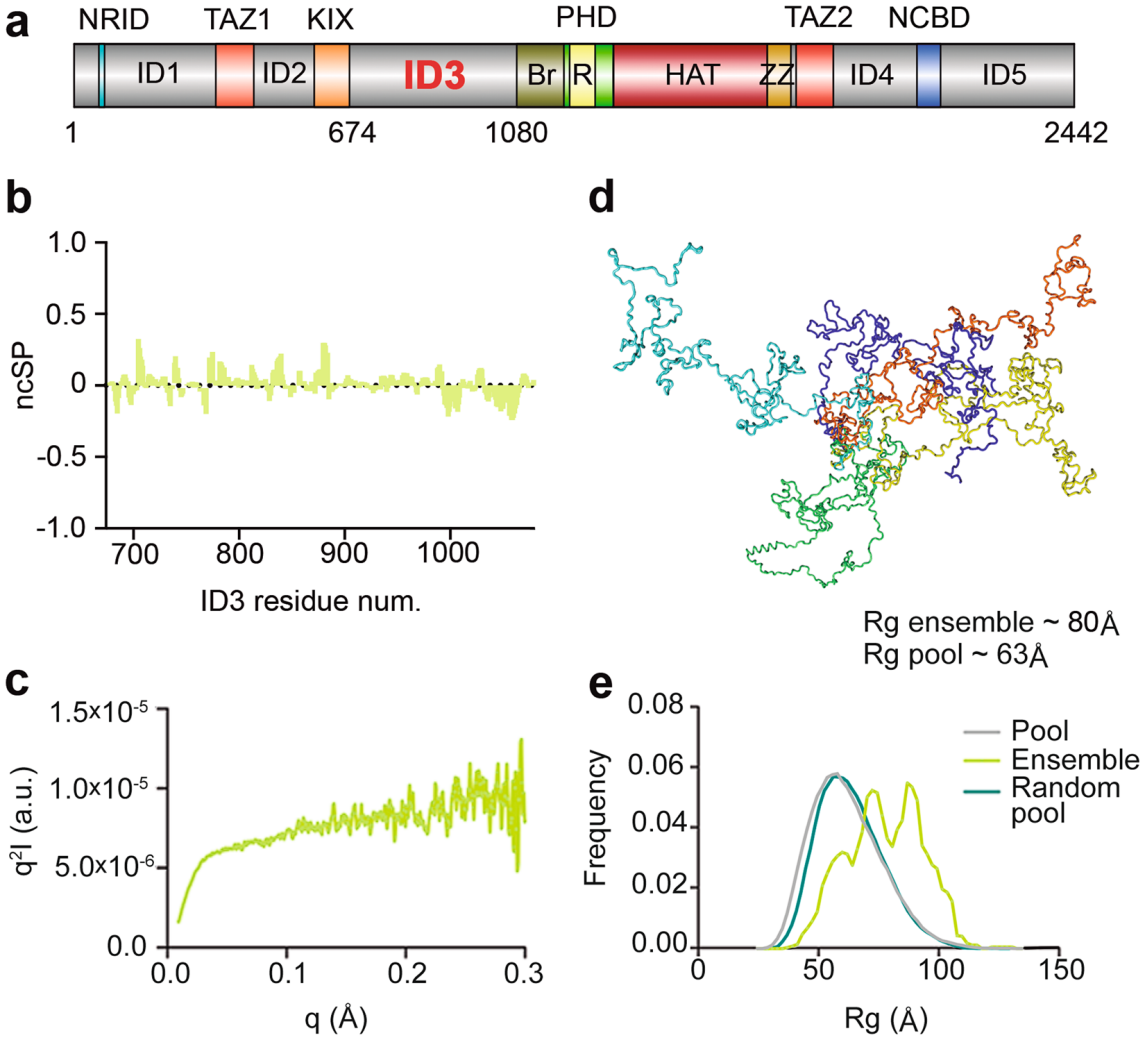
Domain 3 (ID3) of CBP is fully disordered. (**a**) CBP domain organization. CBP globular domains (colored; *Br: Bromodomain, R: Ring*) are connected by intrinsically disordered regions (IDRs, grey) of different lengths. The intrinsically disordered region 3 (ID3, aa674–1080) is located between the KIX domain and bromodomain of CBP. (**b**) Neighbor corrected structural propensities (ncSP) obtained from experimentally measured N, C′, C^α^ and C^β^ chemical shifts. Positive and negative values correspond to α-helical and β-sheet propensities, respectively. (**c**) SAXS Kratky plot also shows an overall disordered state, with a minor tendency for local secondary structure formation. (**d**) Ensemble of selected conformations ID3 may adopt in solution. (**e**) Rg distributions of a random pool of ID3 conformations (teal), of the pool of ID3 conformations biased by experimental ncSP scores (grey) and the ensemble calculated by Flexible Meccano and selected by GAJOE (green).

CBP/p300 are about 2400 residues in length and contain several highly conserved domains and motifs, such as zinc-finger motifs PHD, RING, TAZ1, TAZ2 and ZZ, the CREB binding (KIX) domain, nuclear receptor coactivator binding domain (NCBD), bromodomain and HAT domain. Long intrinsically disordered regions (IDRs, Fig. 1a) connect the structured domains that mediate interactions with more than 400 partner proteins^1^. Although intrinsically disordered proteins/regions of proteins (IDPs/IDRs) have frequently been implicated in protein-protein interactions^5^, the function(s) of the IDRs in CBP/p300 has never been investigated in detail. Rather, it has been suggested that their role is to “connect” the folded, functional elements, enabling CBP/p300 to engage with distinct patterns of chromatin-bound transcription factors in assembling large signaling complexes, such as the enhanceosome^6^. However, it would be quite surprising if about half of the protein primary sequence would only have the function of “connecting” globular functional domains, and therefore it is likely that a variety of different unexplored functional roles are encoded in those long disordered regions. The N- and C-terminal IDRs in CBP/p300 may also enable long-range structural/functional communication within the protein, as exemplified by the repression of HAT activity by phosphorylation of N-terminal Ser 89 in p300^7^. HAT activity and substrate specificity are also regulated by the flanking domains of HAT (bromodomain, PHD, RING, ZZ and TAZ2 domain) through intramolecular interactions^8^.

To assess the possible binding and regulatory role(s) of IDRs in CBP/p300, we are conducting systematic studies on the structural state and binding function of the IDRs of CBP, including ID3 (located between the KIX domain and bromodomain), ID4 (located between the TAZ2 domain and NCBD domain)^9^ and ID5 (located at the C-terminal end of the molecule, past the NCBD domain; cf. Fig. 1a). Our ultimate aim with these studies is to develop a comprehensive structural-functional model of CBP/p300^9, 10^, that also includes the role(s) of IDRs and their interplay with globular domains. No specific interaction has been mapped to any of these regions yet^6, 11^. For ID3, we identified several potential new interaction partners in yeast two-hybrid (Y2H) screenings, among them the Zinc-finger protein 106 (ZFP106). Recent studies suggest that this nuclear protein, ZFP106, is involved in RNA processing and it has been associated with neuromuscular and neurodegenerative disorders^12–15^ such as amyotrophic lateral sclerosis (ALS). We identified ZFP106 as a novel substrate of CBP-mediated acetylation. Active, full-length CBP acetylated ZFP106 on several lysines residues located in two main clusters. The transient interaction between the two IDRs was confirmed by a combination of competition-binding assays, cross-linking experiments and NMR spectroscopy. Finally, we determined the regions of ID3 and ZFP106 involved in the fuzzy interaction by employing paramagnetic relaxation enhancement (PRE) NMR spectroscopy.

## Results

### ID3 is fully disordered

Although bioinformatics predictions suggest that ID3 is an IDR, its structural properties have never been experimentally characterized. To this end, we carried out the structural characterization of the domain by several techniques in reducing buffer at physiological salt concentration. Both in SDS-PAGE and size-exclusion chromatography (SEC), the apparent molecular weight (Mw) of ID3 is about 70 kDa, two times its actual Mw, which is often the first indication of structural disorder^16^. In far-ultraviolet (UV) circular dichroism, ID3 has a strong negative peak around 200 nm, typical of random coil proteins, with a slight tendency to local secondary structure formation^16^ (Supplementary Fig. S1b). Lack of long-lasting structural elements in ID3 allowed the employment of a heat-treatment step^17^ in the protein purification protocol, which increases the purity and stability of the sample. This was confirmed by comparing circular dichroism and 2D ^1^H-^15^N NMR spectra of ID3 obtained with and without this step (cf. Supplementary Fig. S1b and S1c), no significant difference between the two samples could be observed. Solution NMR has become the preferred technique for studying IDPs/IDRs^18, 19^ as it allows a detailed structural analysis of the polypeptides. To achieve the essentially complete sequence specific assignment (about 95%) of the 407 residues of ID3, including its 75 prolines (18%), the recently developed NMR strategy to study complex IDPs, which combines ^1^H and ^13^C detected high-resolution multi-dimensional NMR experiments^18, 20–22^ was necessary (Supplementary Fig. S1a and Tables S1 and S4). The chemical shift (CS) assignment of this disordered domain was facilitated by acquiring additional experiments on a shorter N-terminal fragment of ID3 (ID3-F1 from residue 674–848), which were used also to validate the assignment of the full-length linker. NMR was also used to characterize ID3 backbone dynamics by measuring ^15^N *R*_*1*_, ^15^N *R*_*2*_ relaxation rates and heteronuclear ^1^H-^15^N NOE values (hetNOE) (Supplementary Fig. S2). The domain showed high local mobility on the picoseconds to nanoseconds timescale within residue 725–750 and 775–800, displaying higher ^1^H-^15^N hetNOE and ^15^N *R*_*2*_ values, which indicate slightly more structured conformations than in the rest of the polypeptidic chain. Furthermore, the scattered ^15^N *R*_*2*_ values for some residues in the N-terminal region indicate the occurrence of some exchange processes^18^. Secondary-structure propensities (ncSP)^23^ calculated from experimental CS values^24^ suggested short regions of isolated transient α-helices (in the N-terminal half; approximate residue 674–874) or extended β-type conformations (in the C-terminal third; approximate residue 974–1080), highlighting the presence of potential binding sites (Fig. 1b).

The structural properties are different from those of ID4^9^, for which two segments showing significant α-helical propensity were detected, indicating that each linker has specific structural and dynamic properties that are likely to have an impact on their function.

SAXS measurements [following removal of potential aggregates by high-pressure liquid chromatography (HPLC)] yielded an average radius of gyration (Rg) of 80 Å of the different conformers in solution (Fig. 1c and e), corresponding to a disordered state.

### Ensemble description of ID3 structure

To investigate potential pre-formed structural elements in ID3^25^, we combined data from SAXS and NMR experiments and calculated an ensemble of possible conformations that ID3 can adopt in solution. To this end, Flexible-Meccano was used with NMR-derived ncSP (Fig. 1b) as constraints to generate a random semi-pool of 9876 conformers^26^. For every conformer, theoretical SAXS scattering curves were calculated, compared to the experimental SAXS data and used for selecting the best fitting ensemble^27^ (Fig. 1d; for the fit between the experimental SAXS data and the selected pool, see Supplementary Fig. S3a). We observed a preference in the ensemble for more extended conformers, with a shift towards higher Rg values (Fig. 1e). Short dispersed α-helices observed in the ensemble (Fig. 1d) correspond to secondary structure elements derived from the ncSP input (Fig. 1b).

### Potential binding motifs in the disordered ensemble of ID3

In order to highlight the potential role of ID3 in mediating protein-protein interactions, we also analyzed characteristic features of the sequence (Supplementary Fig. S4). In general, about half the residues of ID3 fall into potential binding sites predicted by ANCHOR^28^ and about half the sequence is highly conserved by DisCons^29^, suggesting the presence of multiple interaction sites within this disordered region of CBP. Several moderately populated helical regions in the N-terminal half appear as potential preformed binding sites, e.g. the highly conserved helical segment around residues 824–844 is predicted as a potential molecular recognition feature (MoRF). C-terminal regions of β propensity are highly conserved and have a conspicuous enrichment of post-translational modification (PTMs) sites, suggesting functional regulation of this region. The DynaMine^30^ profile also shows peaks in this region (Supplementary Fig. S4, underlined), which together predispose this region for mediating interaction(s) probably not based on induced folding^31^.

### ID3 potentially interacts with several proteins

**Figure 2:**
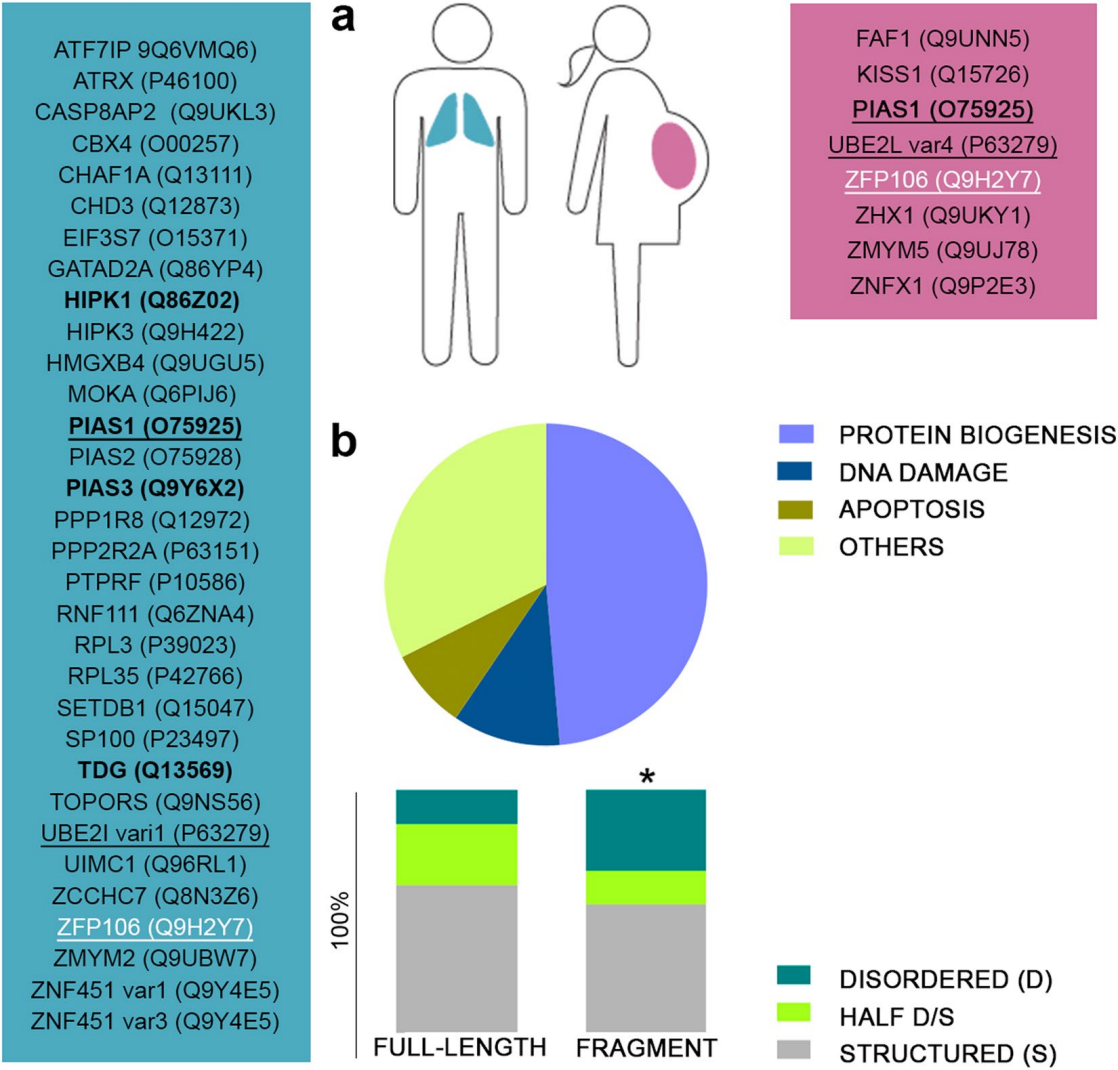
ID3 interacts with the transcription factor ZFP106. (**a**) Potential interaction partners of ID3 have been identified by a yeast 2-hybrid (Y2H) screen. Y2H interactions were classified according to the libraries in which they were detected: placenta (pink), lung cancer (blue), or both (black underlined). Four proteins have been described in previous studies to interact with one or more of the CBP’s globular domains^32–35^ (bold). ZFP106, selected for further studies, is marked in white. (**b**) Potential interactors were analyzed for their structural content (Structured: more than 60% of its sequence predicted to be structured; Disordered: more than 60% of its sequence predicted to be disordered; Half D/S: the rest; *Statistically significant preference of ID3 for binding disordered regions) and for their function. Disorder predictions were carried out with the IUPred webserver^70^, whereas gene ontology (GO) annotations and available literature were used to classify the proteins in four main groups: *Protein biogenesis* (including transcription, pre-mRNA maturation and protein biosynthesis), *DNA damage, Apoptosis* and *Others*.

To find out if ID3 mediates protein-protein interactions, we applied a Y2H screening on two different cDNA libraries, one from human placenta (important for its broad spectrum of expressed genes) and another from human lung cancer (useful to observe potential disease-related interactions). We expected that besides general, housekeeping proteins, shared partners in the two different libraries may point to functionally important and potentially disease-associated interaction partners of CBP/p300 mediated by ID3, mitigating the effect of known high false positive hit rates of Y2H. A total of 37 proteins were classified as potential ID3 binders, including 4 candidates that have already been described to interact with CBP or p300 (HIPK1^32^, PIAS1^33^, PAIS3^34^ and TDG^35^) (Fig. 2a). Surprisingly, sequence analysis of the interacting fragments and full-length proteins highlighted a preference of ID3 for binding disordered regions within interacting partners (Fig. 2b; for the statistical test indicating significance preference, see Supplementary Fig. S3b). Importantly, about half of the identified protein partners were involved in processes related to protein biogenesis, such as gene transcription, pre-mRNA maturation and protein biosynthesis. Among those, we decided to further investigate the transcriptional regulator ZFP106 (Q9H2Y7), because its role in immune response, muscle differentiation, DNA damage, splicing and motor and sensory neuron survival^14, 36^. Further, ZFP106 and CBP may be co-localized in nuclear speckles and be part of a larger complex regulating mRNA production or maturation^12, 37, 38^. ZFP106 was identified in both libraries and structural disorder is involved in recognition in both binding partners. In the placenta library, residues 1265–1521 of ZFP106 were identified, whereas a second, overlapping fragment (residue 1007–1448) was found in the lung cancer library (this clone was frame-shifted, which is probably compensated by translational frame-shift in yeast). Only one copy was found in each library, which is consistent with the low expression levels of ZFP106 in the respective tissue types^38^. For the detailed characterization of the ID3 – ZFP106 interaction, the shorter fragment 1265 to 1521, denominated as ZFP106-f, was selected.

### The transcriptional regulator ZFP106 is acetylated by full-length CBP

**Figure 3:**
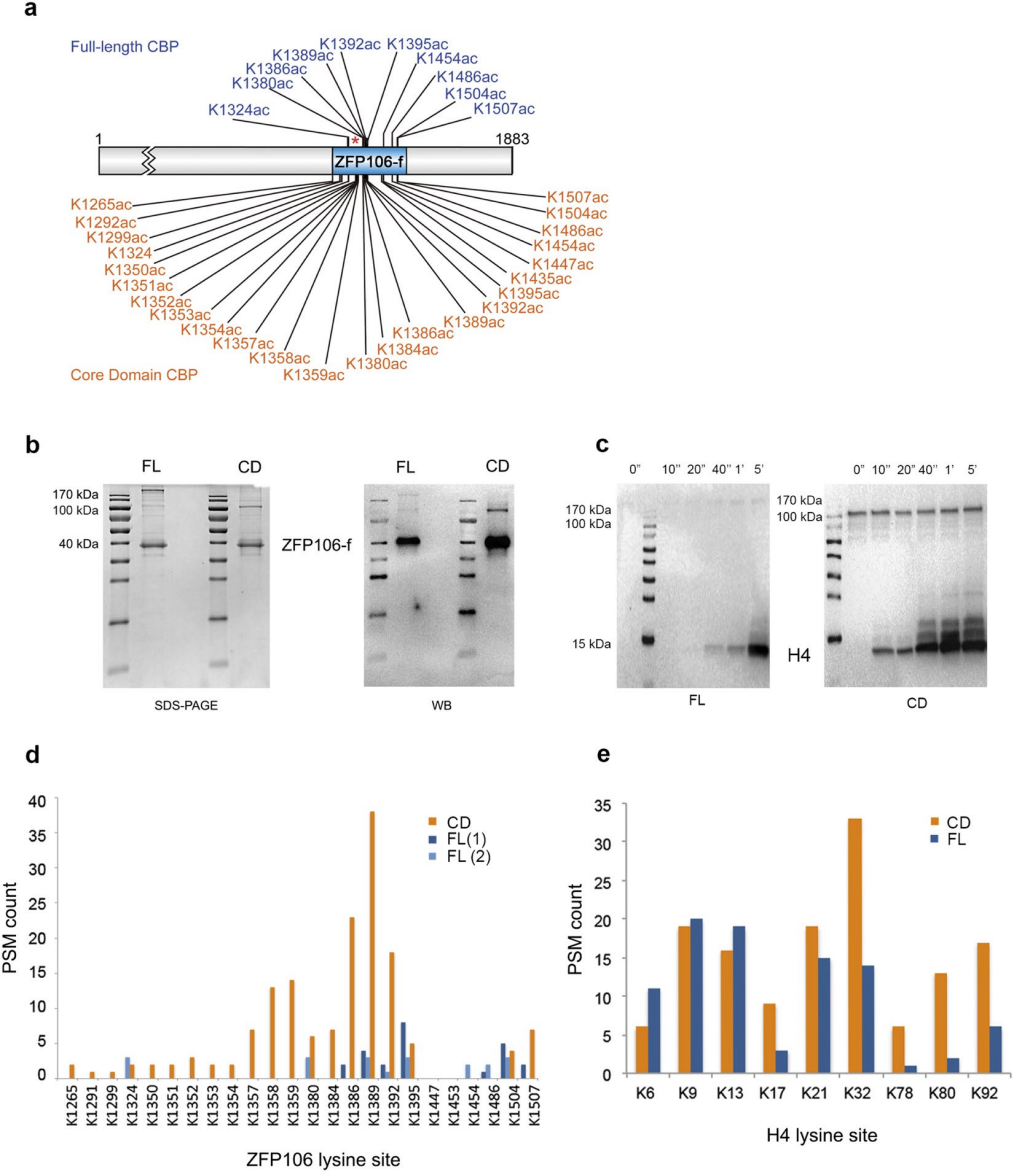
ZFP106 is specifically acetylated only by full-length CBP. (**a**) ZFP106-f was acetylated by either full-length (FL) or the catalytic core domain (CD) of CBP under comparable conditions, and the sites of acetylation were determined by MS. Specific acetylation pattern by full-length CBP (blue) is in contrast to the indiscriminate acetylation achieved by the core domain (orange). *Location of the lysine rich patch of ZFP106-f. (**b**) SDS-PAGE and WB developed with anti-acetylated-lysine antibody of ZFP106-f acetylated by the full-length or core domain of CBP. (**c**) Western blot developed with anti-acetylated-lysine antibody of histone 4 (H4) acetylated by the full-length or core domain of CBP. Time points of the reaction are presented and highlight a much higher activity for the CD compared to the FL enzyme. (**d** and **e**) The ratio of acetylated peptides corresponding to various lysines identified by MS using ZFP106-f (**d**) or H4 (**e**) as a substrate, acetylated with full-length CBP (dark blue: first MS analysis; light blue: second MS analysis) or its core domain (orange).

CBP/p300 have recently been reclassified as lysine acetyltransferase 3 (KAT3) that can acetylate interacting protein partners, primarily transcription factors or coactivators^1^. To probe such a functional consequence of the interaction between CBP and ZFP106, we assayed the acetylation of ZFP106-f with active, full-length CBP. The reaction was performed in HAT assay buffer in the presence of reducing agents, at low protein concentrations. Western blot (WB) analysis with anti-acetylated-lysine antibody showed a band for the 50 kDa ZFP106-f, revealing ZFP106 as a novel substrate of CBP (cf. Fig. 3).

By MS, no basal acetylation (prior to CBP addition) of the ZFP106-f sample was detected, whereas CBP targeted lysines primarily within two main hot-spots in the C-terminal half of the protein, acetylating only ten lysines out of twenty-four (in about 5–10% of the population) (Fig. 3a). For the complete MS sequence coverage, two MS analyses (FL-1 and FL-2) were needed as each independent analysis covered only 62% and 90% of ZFP106-f amino acid sequence (Fig. 3d).

### Acetylation of ZFP106-f by the CBP core domain alone is efficient but nonspecific

In order to determine if the targeting of lysine acetylation of ZFP106-f required specific protein-protein interaction in the context of full-length CBP, we compared the acetylation pattern of ZFP106-f and histone H4 (the classic CBP substrate) by the core domain (which contains the active HAT domain plus flanking small, globular domains: bromodomain, PHD, RING, ZZ and TAZ2, cf. Fig. 1a) and full-length CBP. Acetylation by the core domain reaches saturation much faster than acetylation by full-length CBP (e.g. H4 acetylation, Fig. 3c). With H4, there are already some differences between the ratio of acetylation of specific (functional N-terminal tail, K6 to K32) and non-functional (apparently non-functional, K78 to K92) lysines by the core domain and full-length CBP, i.e. full-length CBP has some capacity to discriminate between specific and non-specific sites. When ZFP106-f acetylation is studied this discrimination is much stronger; the core domain acetylates all the lysines available, while full-length CBP acetylating only a subset of them (about 10/24, Fig. 3a and d). Thus, the modification by the core domain can be considered indiscriminate, whereas by the full-length CBP “specific”. This can be interpreted as substrate targeting via specific protein-protein interaction between ZFP106-f and full-length CBP, involving its ID3 region.

### Interaction of ID3 and ZFP106 assessed by competition-binding

**Figure 4:**
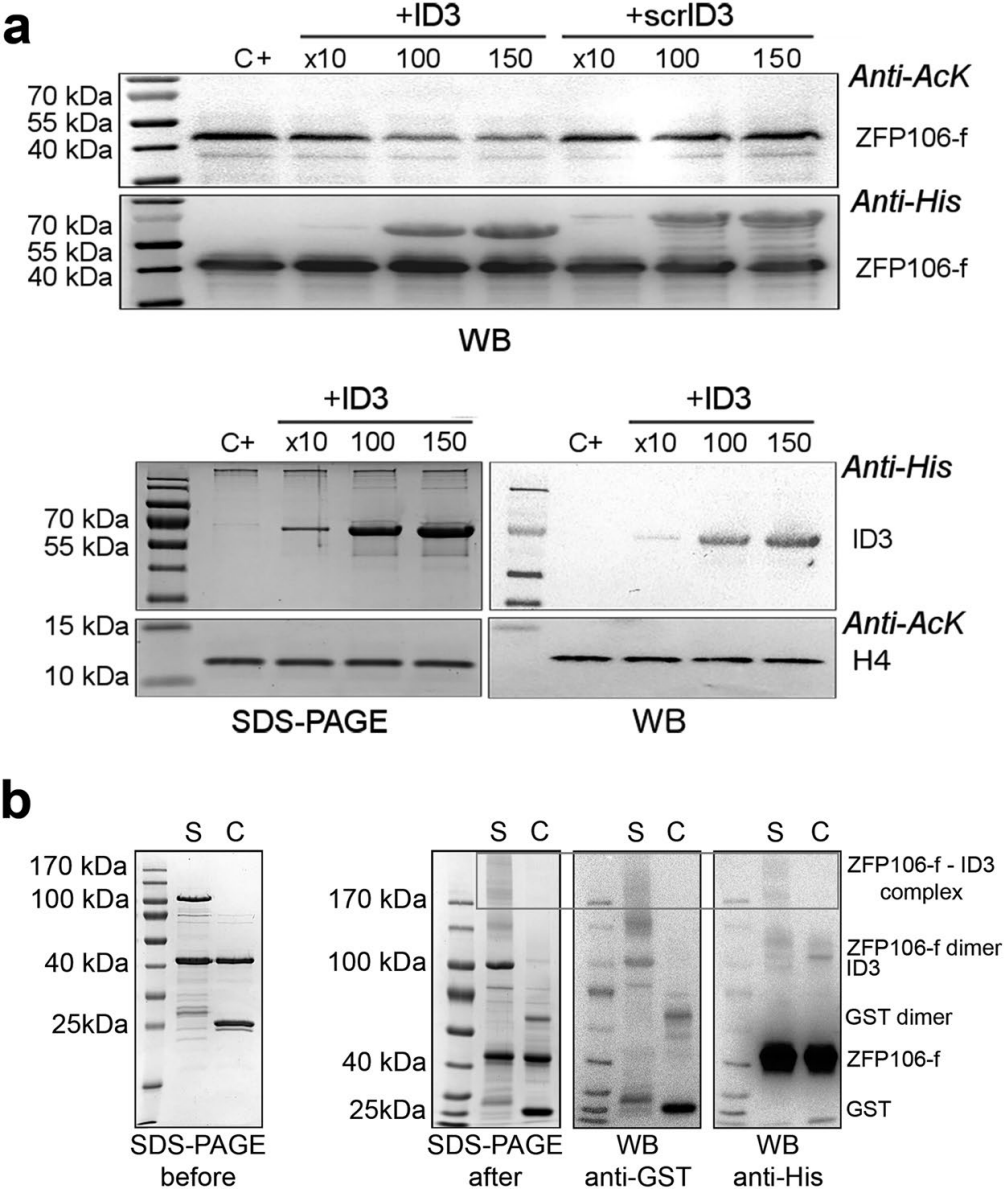
ID3 –ZFP106-f interaction assessed by competition binding assay and cross-linking. (**a**) Top: Competition assays with ZFP106-f as a substrate demonstrate that recombinant ID3 competes with ID3 in the full-length CBP for binding ZFP106-f, which results in a decrease of the ZFP106-f acetylation in the presence of an excess of ID3. However, addition of the same amount of scrambled ID3 (scrID3) has no effect. Bottom: as a control, the same experiment was run using histone 4 (H4) as a substrate; no change in H4 acetylation level could be detected after addition of an excess of ID3. Acetylation was followed by anti-Ac-Lys antibody, adding ID3 to the acetylation reaction in an excess of 10, 100 or 150 times to full-length CBP (18.5 pmol) and its effect was also compared to that of scrambled-ID3 (scrID3; control reaction) *(C*+: *control reaction)*. (**b**) Cross-linked ZFP106-ID3 complex (S: sample reaction) is shown by the presence of high-Mw overlapping bands in the SDS-PAGE gel after cross-linking, anti-His WB and anti-GST WB (box). Under similar condition, ZFP106-GST (C: control reaction) were not cross-linked. Cross-linked complexes formed during the reaction are detailed on the right side of the WB and they have been determined by comparing signals between the anti-His and anti-GST WB (GST 25 kDa; ZFP106-f 40 kDa; GST dimer 62 kDa; ID3 100 kDa, ZFP106-f dimer 115 kDa, ZFP106-f – ID3 complex above 170 kDa).

The discovery of ZFP106-f as a novel substrate of CBP-mediated acetylation allowed us to ascertain the interaction of ID3 and ZFP106-f via competition-binding assays. To investigate the interaction in more detail, we carried out the competition assay by titrating recombinant ID3 into the acetylation reaction with active, full-length CBP. The free ID3 was found to compete with the ID3 of full-length CBP for binding the substrate, ZFP106-f, causing a significant concentration-dependent decrease in the acetylation of ZFP106-f (Fig. 4a). To provide evidence for the specificity of the interaction, we repeated the competition assays with an excess of ID3 of “scrambled” sequence, scrID3 (maintaining the original amino acid composition), which is an approach often used to provide evidence for the specificity of interactions of IDPs^31^. Scrambling abrogates all possible sequence-specific interaction, while maintaining the ones that only derive from physical features relying on amino acid composition. In addition, we generated an ID3 deletion mutant lacking the C-terminal region (ID3delCTR) (Supplementary Fig. S5), since this region was found to interact with ZFP (cf. section “Dissecting the fuzzy ID3-ZFP106-f interaction”). This construct also fails to have an impact on the acetylation level of ZFP106-f (Fig. 4a and Supplementary Fig. S5). Furthermore, in another control experiment with H4 as a substrate, ID3 did not induce a reduction of H4 acetylation, excluding the possibility that ID3 might directly inhibit CBP-HAT activity or bind to another CBP domain and alter its catalytic activity (Fig. 4a and Supplementary Fig. S5). Overall, these results show that ID3 specifically binds to ZFP106-f via defined sequence features.

### Fuzzy interaction of ID3 with the transcriptional regulator ZFP106

**Figure 5:**
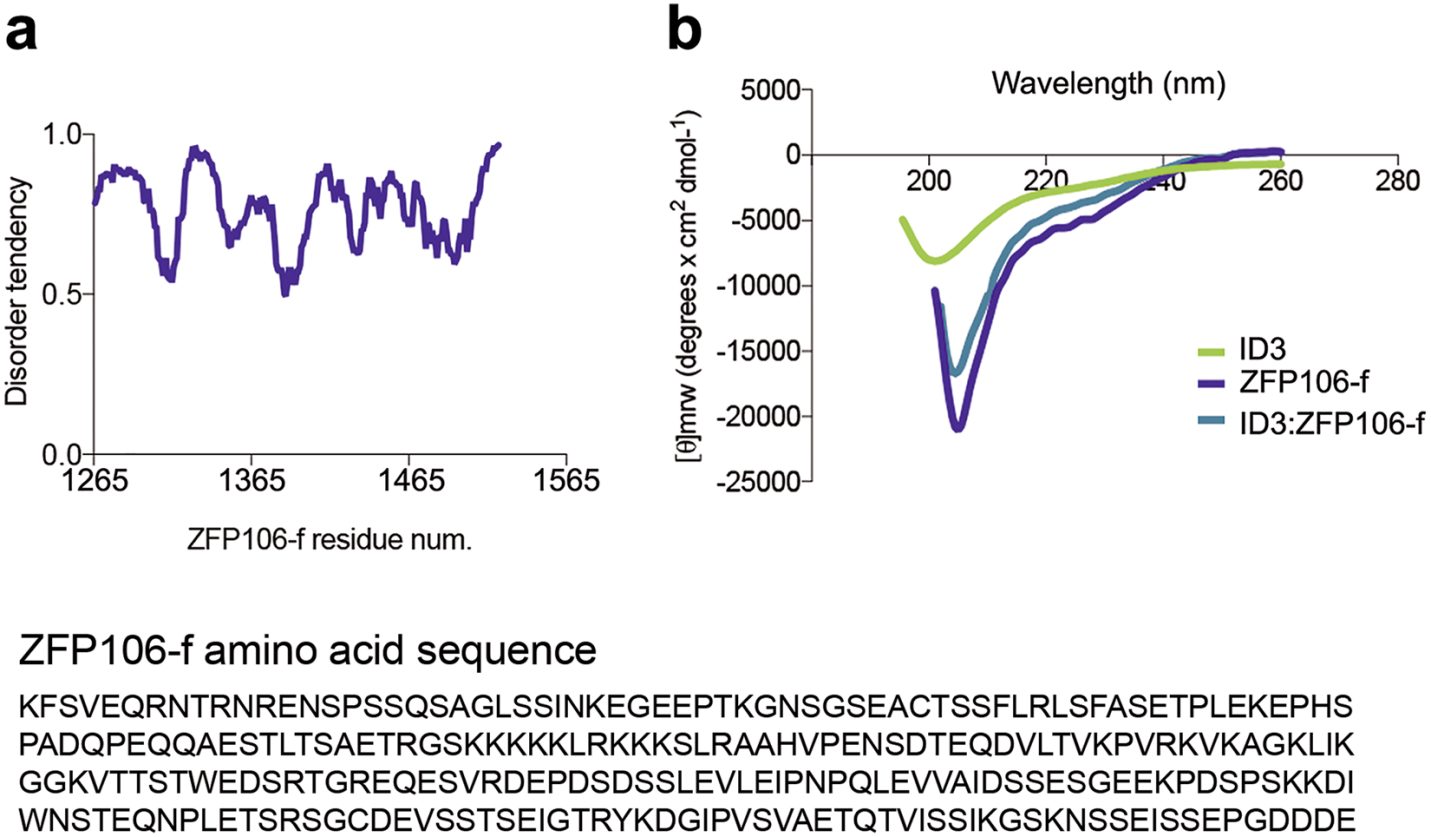
Fuzzy interaction between the two disordered regions. (**a**) Top: Disorder prediction of ZFP106-f using IUPred server indicates a high disorder tendency. Bottom: The ZFP106-f amino acid sequence used for the disorder prediction. (**b**) Circular dichroism spectra of ID3, ZFP106-f and 1:1 ratio of ID3:ZFP106-f. The spectrum of the complex resembles the individual spectra indicating no major changes in the structural content of the two partners upon association.

*In silico* analysis and circular dichroism experiments (Fig. 5) showed that ZFP106-f is also largely disordered, which suggests a rather transient binding between the two IDRs that can occur either via a mutual (synergistic) folding^39^ or a “fuzzy” interaction^31^, in which both IDRs remain largely disordered. In fact, Y2H experiments are particularly suitable for detecting transient protein interactions^40^, whereas in co-IP and SEC experiments no strong or stable interaction was detected. Circular dichroism measurements confirmed the fuzziness of the complex, in which neither of the partners undergoes major structural rearrangements upon association (Fig. 5b). Next, we applied cross-linking with BS3 (bis(sulfosuccinimidyl)suberate)^41^ to capture the transient interaction of GST-tagged ID3 (or free GST as control) and His-tagged ZFP106-f (Fig. 4b). We observed on SDS-PAGE the formation of high-Mw complexes in which the presence (and thus interaction) of both protein partners was proven by two consecutive Western blots (with a membrane stripping step in between) with anti-His and anti-GST antibody to visualize high-Mw overlapping bands (box). It should be noted that in these experiments the Mw of cross-linked bands does not correspond to the sum of the Mw of the individual proteins, because cross-linked protein products migrate anomalously on the gel, as previously described^42^. In addition, ZFP106-f appears to be intermolecularly crosslinked to form homodimers of higher apparent Mw (about 115 kDa for a dimer of 80 kDa), and also intramolecularly, in multiple different ways, causing the band to appear as a smear of heterogeneous apparent Mw population (as a consequence of the high number of lysine residues in the protein). Specificity of the cross-linking was assessed in our control reaction (C), where under the same conditions no complex formation between GST and ZFP106-f could be observed (Fig. 4b and Supplementary Fig. S6).

### Dissecting the fuzzy ID3 - ZFP106-f interaction

To identify the residues of ID3 involved in the interaction with ZFP106-f, 2D ^1^H-^15^N NMR spectra of ID3 were recorded in the presence of increasing amounts of ZFP106-f. Only very minor and dispersed chemical shifts (CS) perturbations could be observed along the ID3 sequence (Supplementary Fig. S7a). Given the other lines of evidence for the interaction of the two proteins, this can be interpreted in terms of a fuzzy interaction with dispersed and heterogeneous interfaces between the two proteins. CS is not sensitive to this type of interaction, because measured CS values are linear, population-weighted averages of all protein species and conformations.

**Figure 6:**
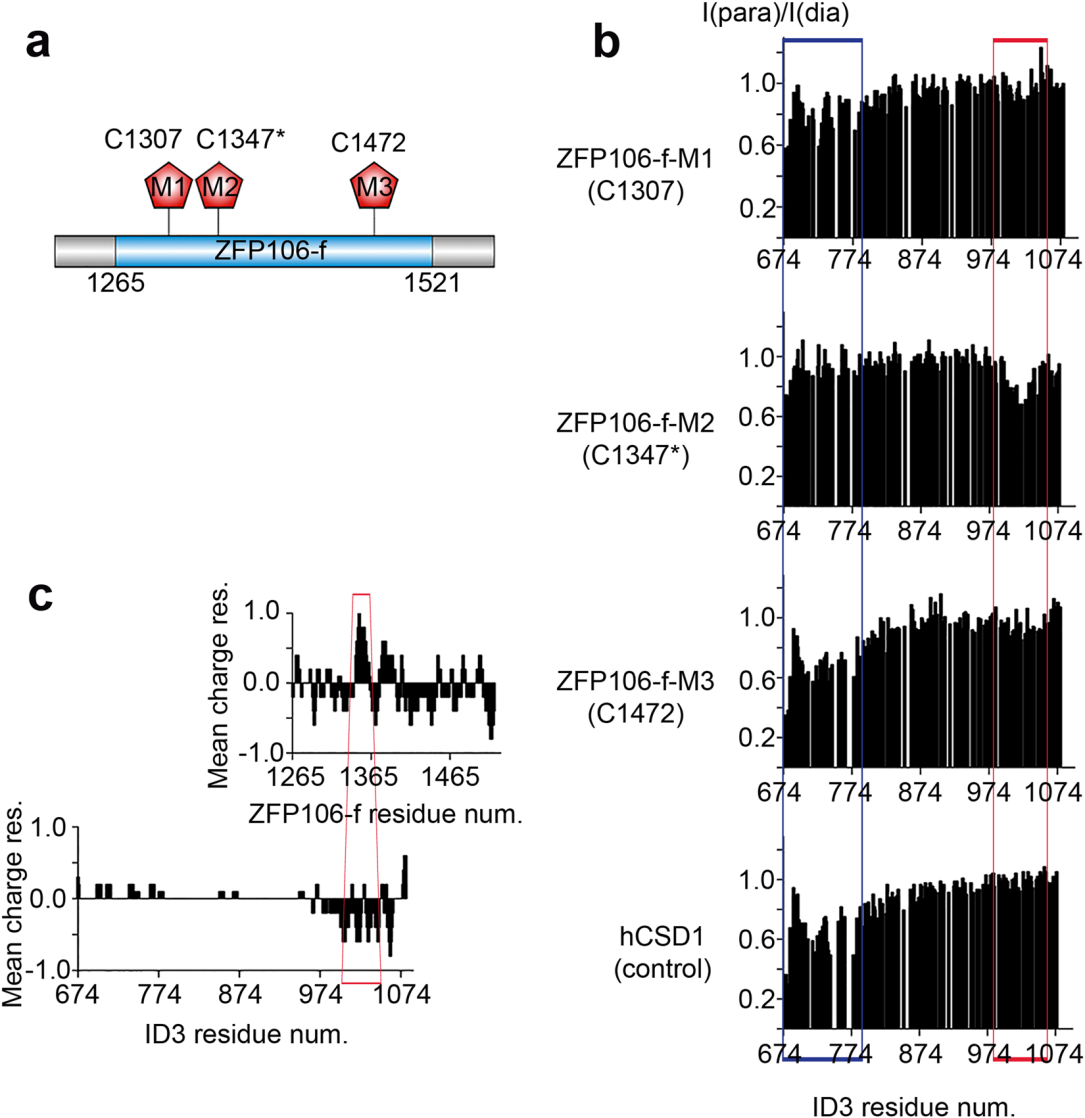
CTR of ID3 specifically interacts with the middle region of ZFP106-f. (**a**) Representation of the ZFP106 fragment identified in the Y2H assay (blue) and the three positions (M1, M2 and M3) where a paramagnetic label was attached to the protein. (**b**) *I*_(para)_ */*I_(dia)_ plots of the 2D ^1^H-^15^N HSQC cross-peaks intensities of ID3 in the presence of the paramagnetically tagged single-Cys ZFP106-f mutants M1, M2 and M3 showing the site of interaction within ID3 (red box). A control experiment with an unrelated IDP, domain 1 of human Calpastatin (hCSD1), labeled with the same paramagnetic probe, is also shown to outline sticky region(s) of ID3 engaged in non-specific interaction (blue box). (**c**) Charge distribution plots of ID3 and ZFP106-f amino acid sequences calculated with the EMBOSS website (http://www.bioinformatics.nl/cgi-bin/emboss/charge). The regions involved in the interaction are highlighted (red): the middle region of ZFP106-f close to the M2 site, and the C-terminal 100 residues (CTR) of ID3.

Therefore, to better characterize the interaction of the two proteins, we turned to paramagnetic resonance enhancement (PRE) measurements. By paramagnetically tagging one of the interaction partners, residues close to the interaction site of the second protein can be detected by decreases in signal intensity. Because PRE effects are averages weighted by the <r^−6^> -dependence of the paramagnetic effect, even a minor population with shorter distance between the nuclei and the paramagnetic probe (r) can have a larger - and thus observable - contribution to the signal measured^43, 44^. To outline the ID3 and ZFP106 regions involved in the interaction, PRE NMR experiments were carried out by labeling ZFP106-f at three different positions (M1, M2, M3) with an EDTA(Mn^2+^)-based paramagnetic tag (Fig. 6a) (for the diamagnetic references, wt ZFP106-f was used for the entire set of experiments).

The PREs observed with the three ZFP106-f constructs confirmed the interaction and highlighted two significantly affected ID3 regions, one in the N-terminal region (NTR, 674–780) and the other in the C-terminal region (CTR, 999–1050) (Fig. 6b). However, interaction with the NTR of ID3 is seen throughout all the PRE NMR experiments, irrespectively of the paramagnetic probe attachment site, and was also observed with an unrelated IDR (control experiment), human calpastatin domain 1 (hCSD1), tagged with the same paramagnetic probe (Fig. 6b and Supplementary Fig. S7e). The very same NTR region is the one mostly affected by chemical exchange as revealed by the high *R*_*2*_ values (Supplementary Fig. S2b). Therefore, we concluded that the interaction with NTR is nonspecific. Further corroboration came with additional control experiments, in which the paramagnetic label free in solution (upon reduction of the disulfide bond between ZFP106-M2-cys and the label) also gave rise to same type of nonspecific signal in NTR of ID3, whereas it did not affect the signal at the CTR, confirming that the observed effects at the CTR of ID3 are due to the specific interaction of the two proteins (Supplementary Fig. S7d).

Moreover, the interacting region(s) of ZFP106-f can be delineated from the different effects the presence of the three ZFP106-f constructs has on ID3 signal intensities in the 2D ^1^H-^15^N NMR spectra (Fig. 6b). A strong signal decrease was observed with the ZFP106-f-M2, and much weaker effect with ZFP106-f-M1. The paramagnetic tag sites M1 and M2 are in relative close proximity to each other (Fig. 6a). In contrast, ZFP106-f-M3 caused no change in signal intensity in the CTR of ID3, suggesting an interaction site between residues 1300–1400 of ZFP106 (Fig. 6).

Interestingly, the region following the M2 label in ZFP106-f is enriched in lysines and carries a positive net charge, whereas the CTR of ID3 is highly negatively charged (Fig. 6c), indicating an electrostatically driven interaction. This was confirmed by NMR experiments performed in the presence of salt, which showed that at 150 mM NaCl the PRE effect was significantly reduced (Supplementary Fig. S7b and S7c). Specificity of the interaction could be concluded, though, from i) a still detectable binding at physiological salt concentrations, and ii) cancellation of the PRE effect on the CTR (but not by NTR) upon the application of DTT, which removes the paramagnetic label from the protein (Supplementary Fig. S7d).

In all, these PRE experiments provided evidence for a transient electrostatic interaction between the two proteins mediated by the negatively charged CTR of ID3 and a positively charged lysine-rich patch in the middle of ZFP106-f.

## Discussion

CBP is a multi-domain transcription coactivator that mediates and integrates the action of hundreds of transcription factors and coactivators, which interact with its globular domains and target its activity on the chromatin and/or other proteins^2, 6^. The function of its long IDRs has been mostly described as that of passive linkers. Here, we focused our attention on possible additional roles of the 407 residues long ID3 located between the KIX domain and bromodomain of CBP.

SAXS, circular dichroism and NMR spectroscopy showed for ID3 a random coil state. By integrating ^1^H-detected and ^13^C-detected high-resolution multi-dimensional NMR experiments^18, 20–22^ we assigned about 95% of backbone chemical shifts, providing atomic resolution information on its structural and dynamic features. An ensemble of possible conformations calculated with experimentally derived ncSP scores and SAXS data supported the presence of several potential binding sites with mild local helical or β propensities, within a largely disordered module. The extended and flexible conformations of ID3 are expected to favor functions/interactions that take advantage of its dynamic properties. In accord, the specific binding role(s) of ID3 is supported by our Y2H studies in two cell-lines that uncovered dozens of potential ID3 binding partners. Among those, the transcriptional regulator ZFP106 was identified as a new acetylation substrate of CBP *in vitro*, which is in line with prior MS-based proteomic studies, which showed acetylation of ZFP106 on some of the lysine residues identified here (cf. PhosphoSitePlus^45^). The interaction of ZFP106-f with ID3 was confirmed by cross-linking and the observed competition between ID3 and full-length CBP in the acetylation reaction. Specificity of the interaction is underscored by the highly nonspecific acetylation of ZFP106-f by the core domain of CBP, which also strongly acetylates the lysine-rich patch located in the middle of ZFP106-f. PRE experiments, at low and high salt concentration, allowed us to determine the ID3 – ZFP106-f interacting regions, which include an extended negative patch of ID3 (roughly, residues S980-P1080) and a mostly positive patch of ZFP106-f (roughly, residues P1340-K1395). These experiments together with the HAT competition assays, in which the C-terminal charged region of ID3 has been deleted (Supplementary Fig. S5) confirmed the electrostatic nature of the interaction. The ZFP106-f lysine-rich patch involved in the interaction with ID3 is not acetylated by the full-length CBP, suggesting that ID3-ZFP106-f interaction may be involved in targeting specific lysine acetylation of ZFP106-f by “hiding/exposing” some ZFP106-f regions from/to the CBP-HAT domain. Specificity of this targeting relation is further corroborated by the similar behavior of H4, the archetypic substrate of CBP/p300. With H4, the core domain cannot distinguish between specific (N-terminal histone tails; K6 – K21) and nonspecific (K32 – K92) acetylation sites, whereas full-length CBP shows a strong preference for the former.

An intriguing aspect of our investigation is the transient character of this targeting interaction; such fuzzy and/or weak and transient complexes have been suggested to confer specificity without a structured state^31, 46^. For example, it has been shown that the weak, high-μM interaction of the cytoplasmic tails of T–cell receptors in T–cell signaling occurs via their disordered state, as suggested by circular dichroism and NMR^47^. The physiological regulatory relevance of an even weaker (“ultraweak”) interaction has been demonstrated in focal adhesion dynamics. Here, the interaction of the SH3 domain of Nck2 and the LIM4 domain of PINCH-1 has a Kd of 3 mM, still it is indispensable in integrin signaling, as suggested by genetic evidence^48^. The importance of transient, lowly-populated states that are still invisible to most structural biology techniques has also been demonstrated in encounter complexes^49^, RNA-protein recognition^50^ and the catalytic cycle of dihydrofolate reductase^51^. However, we cannot exclude the possibility that other domains of CBP or ZFP might be also involved in the interaction and influence affinity. This is also corroborated by the ID3 competition experiments, in which ID3 in a large molar excess (10x, 100x) is required to compete with binding and targeting of ID3 region within full-length CBP.

It is worth mentioning that the identified interaction regions are also rather long and may contain specific interaction motifs or charge distribution required for the interaction. Especially, ID3 sequence presents a characteristic and regular distribution of charges that has been conserved during evolution (*cf.* Fig. 6c and Supplementary Fig. S4). Similar binding features may also be present in long IDRs of other multi-complex proteins, such as bacterial RNAse E^52^, BRCA1^53^, or UPF2^54^. Moreover, the observed acetylation of ZFP106-f fits perfectly with CBP/p300 function in general: CBP/p300 integrate the action of hundreds of transcription factors and coactivators, the function of which is often regulated by acetylation^2, 3^. In this context, the highly significant enrichment of ID3 binding partners for transcription-regulatory and DNA-binding functions is notable^4^. Moreover, “*in vivo*” acetylation of ZFP106 was previously described by Li Y. and colleagues in 2006. High-throughput MS analysis of acetylated proteins present in colorectal carcinoma cell lines (SNU-C2B) identified four acetylation sites in ZFP106 (K1350-ac, K13510-ac, K1352-ac, K1358-ac) (Record ID: 11618 in PhosphoSitePlus^45^) within a region that is comprised in our construct (AA1265–1521). Interestingly, in our *in vitro* experiments those sites seem only to be acetylated by the highly active and nonspecific core domain but not by the full-length CBP.

In a cellular context, ZFP106 and CBP share roles in developmental pathways and diseases such as cancer. They also share a number of interaction partners, such as the tumor suppressors APC (Adenomatous polyposis coli protein) and Smad2 (Mothers against decapentaplegic homolog 2), which suggests that ZFP106 maybe involved in pathways such as TGFβ or Wnt signaling. In all, it seems reasonable that ZFP106 and CBP are part of a larger transcriptional complex that regulates expression of target genes involved in specific cellular processes, such as muscle cell differentiation or maintenance of the neuromuscular signaling^12, 38, 55^. Recent studies have shown that the nuclear ZFP106 protein that localized in nuclear speckles is required for normal assembly of the mammalian spliceosome, a process in which CBP/p300 also seems to participate^12, 14, 56, 57^.

In summary, our results suggest a possible additional role of an IDR in CBP, which may not only lead to a better description of the function of CBP/p300 and ZFP106, but also provides a precedence for addressing the function of long “linker” regions in large multi-domain signaling proteins.

## Materials and Methods

### Sequence analysis

A set of homologous ID3 amino acid sequences of various vertebrate species was assembled to study disorder propensities, sequence conservation, and the presence of potential binding sites. Cf. *SI Materials and methods*.

### Scrambling the sequence of human ID3

For control purposes, a scrambled-ID3 sequence sharing 21% identity with wt ID3 was generated. (Supplementary Fig. S8). *SI Materials and methods*.

### Cloning, mutagenesis and recombinant proteins

Full-length CBP (UniProt id Q92793), core domain of CBP (residues 1095–1849); ID3 of CBP, (residues 674–1080); ID3-F1 of CBP (residues 674–848); and Cys-to-Ser mutant ID3(C845S) obtained by site-directed mutagenesis of Cys845 of ID3, were cloned from the full-length construct. ZFP106-f (residues 1265–1521 of full-length ZFP106 (UniProt Q9H2Y7)). Scrambled-ID3 (scrID3), ID3delCTR and the three single-cysteine mutants of the ZFP106-f were cloned by GenScript (M1: C1472S (paramagnetic label on C1307), M2: insertion of Cys between Thr1346 – Arg1347 (paramagnetic label on insC1347*), M3: C1307S (paramagnetic label on C1472)). *For further details, cf. SI Materials and methods*.

### Yeast two-hybrid screening

Y2H analysis was carried out by Hybrigenics Services S.A and described in *SI Materials and methods*. Identified partners were analyzed with IUPred (disorder level) and classified using GO annotation (biological process).

### Recombinant protein expression, purification and labeling

Full-length (FL) CBP and its core domain (CD) were expressed in Sf9 cells, while the rest of the proteins were expressed in *E. coli*. Purification was achieved by a two- or three-step purification protocol including nickel (and Flag for the CBP constructs) affinity followed by size exclusion chromatography.

During ID3 purification a heat-treatment step of the lysate was done to increase the purity of the protein sample^17^, which was shown not to alter the secondary structure content of the protein (cf. Supplementary Fig. S1).

For the paramagnetic labeling, purified single-cysteine ZFP106-f mutants (M1, M2 and M3) and the hCSD1 (for control experiment) were mixed with the metal-containing probe (thiol-reactive label pEDTA). The reaction was carried out overnight at RT and labeled proteins were re-purified. *For further details, cf. SI Materials and methods*.

### Determination of the paramagnetic labeling by intact mass spectroscopy analysis

Intact protein masses were measured by direct infusion in a microelectrospray ionization ion trap mass spectrometer (LTQ XL). The mass spectra were deconvoluted using the software ProMass Deconvolution. *For further details, cf. SI Materials and methods*.

### Circular dichroism spectroscopy

Far-UV circular dichroism spectra of ID3, ZFP106-f and the complex (1:1 molar ratio) were recorded on a J-715 spectropolarimeter in reducing buffer of physiological salt concentration. *For further details, cf. SI Materials and methods*.

### Small-angle X-ray scattering experiments

ID3 was injected into a size-exclusion column equilibrated with buffer of physiological salt concentration and SAXS data were collected continuously, selected frames corresponding to the main protein elution peak were averaged using FOXTROT^58^ (buffer scattering subtracted), following data processing/analysis using ATSAS suite^59^ and calculation of the radius of gyration by PRIMUS QT^60^. *For further details, cf. SI Materials and methods*.

### Nuclear magnetic resonance experiments (NMR) and data processing

All the experiments and acquisition parameters regarding ID3 sequence-specific assignment, backbone dynamics and, PRE experiments are reported in *SI Materials and methods and* Tables S1–S5.

#### Sequence-specific assignment

All the multidimensional NMR experiments for sequence-specific assignment were performed on uniformly [^13^C,^15^N] labeled ID3 and ID3-F1 in reducing buffer of physiological salt concentration at pH 6.5, with 10% D_2_O added for the lock. The CS assignment of ID3 has been deposited in the *BioMagResBank* (BMRB, http://www.bmrb.wisc.edu) as entry 26841. *For further details, cf. SI Materials and methods*.

#### Backbone dynamics

Backbone dynamics (at 297 K) of uniformly ^15^N-labeled ID3 were studied by using ^15^N relaxation data obtained from 2D ^1^H-^15^N HSQC-edited experiments^61^, carried out using a buffer of physiological salt concentration at pH 6.5, with 10% D_2_O added for the lock. *For further details, cf. SI Materials and methods*.

#### Paramagnetic relaxation enhancement (PRE) experiments

For the PRE measurements, 2D ^1^H-^15^N HSQC spectra of the paramagnetic and diamagnetic samples were acquired^62^ on [^13^C,^15^N] ID3(C845S) and 1 molar equivalent (eq) of single-Cys ZFP106-f mutant EDTA(Mn^2+^)-tagged (paramagnetic samples). The diamagnetic references were done with wt ZFP106-f with DTT added (wt proteins are equivalent to their EDTA(Zn^2+^)-tagged version for PRE referencing^62^). The unrelated protein control experiments were done with 1 eq of wt hCSD1-EDTA(Mn^2+^) (paramagnetic sample) or wt hCSD1 (diamagnetic sample, DTT added). The reduced-label control experiments, the [^13^C,^15^N] ID3(C845S):ZFP106-f-M2 sample was measured in absence and presence of reducing agents (DTT). All NMR samples were measured at pH 6.5 and 10% D_2_O for the lock, NaCl was added to a final concentration of 150 mM. *For further details, cf. SI Materials and methods*.

#### NMR data processing

NMR data sets were processed using Bruker *TopSpin 1.3* software or *nmrPipe* ^63^. NUS data sets were converted with *nmrPipe* and then processed using either the *Multidimensional Fourier Transform* (MFT) or the *Sparse MFT* (SMFT) algorithm. Both programs are available at http://nmr.cent3.uw.edu.pl. Data analysis was done with *Sparky* ^64^ or *CcpNmr Analysis* ^65^.

### Secondary structure propensity calculation

Secondary structure propensity analysis was calculated from C′, C^α^, C^β^ and N chemical shifts (95% assigned) by using the *neighbor-corrected structural propensity calculator* (ncSP) tool^66^. *For further details, cf. SI Materials and methods*.

### Ensemble modelling

Ensemble models of ID3 were based on the combination of SAXS data and ncSP derived from experimental chemical shift values. Flexible-Meccano^26^ and SCCOMP^67^ were used to generate a pool of 9876 semi-random conformations (with side-chains modeled). Then, CRYSOL^27^ and the GAJOE algorithm were employed^27^ to select subsets of the random pool that that agreed with the experimental SAXS data. *For further details, cf. SI Materials and methods*.

### Acetylation Assay

*In vitro* ZFP106-f or H4 acetylation assays were performed in reducing buffer of physiological salt concentration at 20 or 30 °C using FL or CD of CBP. For the HAT competition assay, x10, 100 and 150 molar excess (relative to full-length CBP) of ID3, scrID3 and ID3delCTR were added to the acetylation reactions. The reactions were completed to saturation and the samples were analyzed by Western blot by anti-acetylated-lysine (Bioke) and anti-His (Sigma-Aldrich) antibodies. For the identification of acetylation sites, the proteins in solution were desalted and digested with either pepsin or endoglu-C, and analyzed by LC-MS/MS in a microelectrospray ionization ion trap mass spectrometer (LTQ XL, or Orbitrap XL) as described^68, 69^. *For further details, cf. SI Materials and methods*.

### Cross-linking Assay

Proteins were mixed in approximate 1:1 ratio (concentrations adjusted by SDS-PAGE). Cross-linking reactions were carried out for 15 min at RT in PBS with 1 mM DTT (S: sample, ID3:ZFP106-f; C: control, GST:ZFP106-f). Crosslinker BS^3^ was added to a final concentration of 0.25 mM. *For further details, cf. SI Materials and methods*.

## Electronic supplementary material

Supplementary Information
