# Supplementary material for: Linking functions: an additional role for an intrinsically disordered linker domain in the transcriptional coactivator CBP
Source: Sci Rep. 2017 Jul 5;7:4676. doi: 10.1038/s41598-017-04611-x (PMC5498717; doi:10.1038/s41598-017-04611-x)
Supplement: Supplementary file 1 — Supplementary Information [file 41598_2017_4611_MOESM1_ESM.pdf]

SUPPLEMENTARY INFORMATION

Linking functions: an additional role for an intrinsically disordered linker domain in the transcriptional coactivator CBP

Sara Contreras-Martos[1,2] & Alessandro Piai[3,4], Simone Kosol[1,2,5], Mihaly Varadi[1,2,6], Angela Bekesi[1,2], Pierre Lebrun[1,2], Alex Volkov[1,2], Kris Gevaert,[7,9] Roberta Pierattelli[3,4], Isabella Felli[3,4], Peter Tompa* [1,2,7]

[1]*VIB-VUB Center for Structural Biology, Vlaams Instituut voor Biotechnologie (VIB), Brussel, Belgium*

[2]*Vrije Universiteit Brussel (VUB), Brussel, Belgium*

[3]*Magnetic Resonance Center, University of Florence, Florence, Italy*

[4]*Department of Chemistry "Ugo Schiff", University of Florence, Florence, Italy*

[5]*Department of Chemistry, University of Warwick, Gibbet Hill, Coventry, CV4 7AL, United Kingdom.*

[6]*Protein Data Bank in Europe, European Molecular Biology Laboratory, European Bioinformatics Institute (EMBL-EBI), Wellcome Genome Campus, Hinxton, Cambridge, CB10 1SD, UK*

[7]*VIB-UGent Center for Medical Biotechnology, Ghent, Belgium*

[8]*Institute of Enzymology, Research Centre for Natural Sciences of the Hungarian Academy of Sciences, Budapest, Hungary*

[9] *Department of Biochemistry, Ghent University, Ghent, Belgium*

** Corresponding author*

To whom correspondence should be addressed: Prof. Peter Tompa, Structural Biology Research Center, Vlaams Instituut voor Biotechnologie (VIB) at Vrije Universiteit Brussel (VUB), Pleinlaan 2, 1050 Brussel, Belgium, Telephone +32 2 629 19 62; E-mail: *peter.tompa*@vub.be

SI MATERIALS AND METHODS

Sequence analysis

PSI-BLAST[1] search was performed using the human ID3 fragment sequence against the non-redundant (nr) protein dataset of NCBI. Multiple sequence alignment (MSA) of ID3 sequences was generated by MAFFT[2] to characterize the conservation of the amino acid sequence. Structural disorder was analyzed using the DisCons online tool[3]. Within DisCons, IUPred in 'short' running mode[4] and VSL2[5] were used to predict residue-level structural disorder; other parameters were left as default. Secondary-structure propensities were predicted by PsiPred[6]. Identification of functional motifs and potential binding sites within ID3 was based on ELM database[7], MoRFpred[8], ANCHOR[9] and DynaMine[10].

Scrambling the sequence of human ID3

To ensure that the scrambled sequence generated is disordered, we used IUPred[4], ESpritz[11] and VSL2[5], and only sequences in which the disorder scores of each residue exceeded 0.5 by all three methods were retained for further analysis. Aggregation-prone sequences were filtered out by TANGO[12] applying a threshold of 0.001. Finally, sequences were also screened for the presence of potential binding motifs by definitions derived from the ELM database, and only sequences without recognizable motifs were retained. Only one protein sequence was selected for the control experiments. ID3 and scrambled-ID3 (scrID3) sequences were aligned using T-Coffe (default mode)[13] and EMBOSS Needle[14]. To graphically display the sequence alignment, BoxShade open service from th**e** SIB ExPASy Bioformatics Resources Portal was used (cf. Supplementary Fig. S8).

Cloning and mutagenesis

Full-length human CBP (UniProt id Q92793) was amplified by PCR from synthesized CBP cDNA (Origene - RC219036), cloned in pDONOR221 and transferred to pDEST10 using Gateway® Cloning Technology (Life Technologies). The gene fragment encoding the disordered domain ID3 (residues 674-1080 of CBP) was sub-cloned into pet16b using the restriction sites *NdeI* and *BamHI* and into pGEX4T using the restriction sites *BamHI* and *XhoI*. The cDNA encoding for a shorter fragment, denoted as ID3-F1 (aa 674-848), was sub-cloned into pet200D via TOPO cloning. The cDNA encoding for the core domain of CBP (residues 1095-1849) was sub-cloned into pDEST10 via TOPO cloning.

The fragment of ZFP106 (ZFP106-f) identified in Y2H binding assay (residues 1265-1521 of full-length ZFP106 (UniProt Q9H2Y7)) was PCR-amplified and cloned into pet16b using the restriction sites XhoI and BamHI. All the constructs were sequenced before transformation into expression strain *E. coli* BL21(DE3).
Cys-to-Ser mutant ID3(C845S) was obtained by site-directed mutagenesis of Cys845 of ID3 following the manufacturer's protocol of QuikChange II Site-Directed Mutagenesis Kit (Agilent #200523).

Scrambled-ID3 (scrID3), ID3delCTR and the three single-cysteine mutants of the ZFP106-f were cloned into pET16b (GenScript, Piscataway, USA) (M1: C1472S (paramagnetic label on C1307), M2: insertion of Cys between Thr1346 – Arg1347 (paramagnetic label on insC1347*), M3: C1307S (paramagnetic label on C1472)).

Yeast-two-hybrid screening

The coding sequence of CBP ID3 (aa 674-1080) (GenBank accession number gi: 119943103) was provided to Hybrigenics Services S.A. The sequence was PCR-amplified and cloned into pB27 as a C-terminal fusion to LexA (N-LexA-ID3-C) and into pB35 as a C-terminal fusion to Gal4 DNA binding domain (N-Gal4-ID3-C). The constructs were confirmed by sequencing and were used as baits to screen random-primed human placenta and lung cancer cDNA libraries. In case of the LexA construct, 142.7 million interactions were analyzed, whereas for the Gal4 construct the number of interactions was 72.2 million. After selection on a medium lacking leucine, tryptophan and histidine, 189 clones were finally processed. The prey fragments were amplified by PCR and sequenced for identification of the corresponding interacting proteins. Sequence analysis of the identified proteins (full length and fragments) was performed using IUPred[15] for disorder prediction and UniProt[16] for gene annotation (GO) mapping. In order to compare the disorder content of the Y2H assay fragments with that of their corresponding full-length sequences, we have assembled two sequence sets. The first set contained the amino acid sequences of all the identified fragments, while the second set consisted of the full-length protein sequences retrieved from UniProt/SwissProt[16]. We used IUPred[15] to quantify the per residue disorder propensities of all the collected sequences and calculated the ratio of disordered residues within each sequences, i.e. the ratio of residues that have a disorder propensity of 0.5 or greater. The distribution of these ratios within the two sequence sets was compared (c.f. Supplementary Fig. S3b). The distributions were compared using a non-parametric paired Wilcoxon-Mann-Whitney test, since this statistical test does not rely on Gaussian distribution and only assumes continuity of the variables and independence within the two samples. According to the test the average ratio of disordered residues is significantly higher in the fragments than within the full-length sequences (p-value 0.03808). This indicates that the fragments that were identified by the Y2H assay are relatively more disordered than the rest of their protein chains, and consequently this might suggest that ID3 favors interactions with disordered binding regions.

Recombinant protein expression, purification and labeling

1) <u>Full-length CBP and its core domain</u>

Full-length CBP was expressed in Sf9 insect cells[17] and purified by consecutive nickel (His-select, Sigma-Aldrich) and anti-FLAG (Sigma-Aldrich) immunoaffinity chromatography followed by size exclusion chromatography in the presence of protease inhibitors (2 µg/ml leupeptin, 1 µg/ml bestatin, 1 g/ml pepstatin A, 5 µg/ml E64, and 2 µM 4-(2-aminoethyl)benzenesulfonyl fluoride hydrochloride (AEBSF)). Chelating agents were avoided to keep the Zn-finger domains intact. Purified protein was flash frozen in liquid N_2 and stored at -80°C in storage buffer (50 mM Tris pH 7.5, 150 mM NaCl, 5% glycerol, 50 µM tris(2-carboxyethyl)phosphine (TCEP), and protease inhibitors: 1 µg/ml leupeptin, 0.5 µg/ml bestatin, 0.5 µg/ml pepstatin A, 1 µg/ml E64, and 0.1 µM PMSF).

<u>2) Other protein constructs</u>

ID3, scrID3, ID3delCTR, ID3(C845S), ID3-F1, ZFP106-f, Single-Cys mutants of ZFP106-f (M1, M2 and M3) and human calpastatin domain 1 (hCSD1) were generated as follows:

Transformed *E. coli* BL21(DE3) (invitrogene) cells were grown in Luria Broth (LB) medium at 37°C until optical density (OD) reached 0.6 and protein expression was induced with 1 mM IPTG. For [^{15}N-^{13}C] labeled protein samples, cells were grown to high cell density and centrifuged (15 minutes, 3500 g); pelleted cells were resuspended in M9 medium and the culture was grown for 2.5 h at 37°C before induction[18]. Cell cultures were harvested after overnight expression at 25°C for all ID3 samples or 2h at 37°C for ZFP106-f. The pelleted cells were resuspended in lysis buffer (50 mM KH_2PO_4, 300 mM NaCl, 10 mM Imidazole, 1 EDTA-free protease inhibitor cocktail tablet (Roche), DNase, lysozyme, pH 8.0), sonicated and followed by heat-treatment (85°C, 10 minutes), in case of all ID3 samples[19]. Cell

lysates were cleared by centrifuging at 13000 g for 45 minutes. Subsequent purifications were done by IMAC (HisTrap HP column; GE Healthcare) followed by size-exclusion chromatography (Superdex200 26/600 and Superdex75 26/600; GE Healthcare) in a buffer 50 mM KH_2PO_4, 150 mM NaCl, 1 mM DTT, pH 7.5, complemented by 2 EDTA-free protease inhibitor cocktail tablets (Roche). Following buffer exchange into distilled water (dH_2O), the sample was dried at room temperature (RT) in a vacuum concentrator (ID3) or lyophilized (ZFP106-f) and stored at -20°C. Samples for PRE measurements, ID3(C845S) and single-cysteine ZFP106-f mutants (M1, M2 and M3), were flash frozen in solution after size exclusion and stored at -80°C.

3) Paramagnetic labeling of the ZFP106-f mutants and hCSD1

Purified single-cysteine ZFP106-f mutants (M1, M2 and M3, see "Protein constructs") and the hCSD1 (for control experiment) were incubated with 10 mM DTT for 15 minutes at 30°C followed by buffer exchange into 20 mM Tris-HCl pH 8.0 in a desalting column (HiTrap 5mL desalting, GE Healthcare). The metal-containing paramagnetic probe was prepared by mixing together one molar equivalent of $MnCl_2$ (Sigma-Aldrich) and the thiol-reactive label, N-[S-(2-pyridylthio)cysteaminyl]ethylenediamine-N,N,N',N'-tetraacetic acid, monoamide (Toronto Research Chemicals, #P996250). The desalted protein sample was mixed with a 5-fold molar excess of the metal-containing probe, and the reaction was carried out overnight at RT. The modified proteins were purified by ion-exchange chromatography (HiTrap Q FF, GE Helthcare) using 20 mM Tris-HCl buffer pH 8.0 and, eluted with a linear salt gradient. A single defined peak was eluted at 0.5 mM NaCl concentration. The purified samples were desalted into 50 mM potassium phosphate, 0.02% NaN_3 pH6.5. All buffers were pre-treated with chelating beads (Chelex, Sigma) to remove traces of heavy metals.

Determination of the paramagnetic labeling by intact mass (mass spectroscopy) analysis

Purified proteins were desalted using C18 Spin Columns (Thermo Scientific) and diluted in 50% acetonitrile 0.1% acetic acid. Intact protein mass measurements were performed by direct infusion in a microelectrospray ionization ion trap mass spectrometer (LTQ XL, ThermoFisher Scientific, San José, CA). The parent ions were submitted to in-source dissociation (SID) using the minimal energy to promote efficient declustering of water molecules and salts adducts. The mass spectra were deconvoluted using the software ProMass Deconvolution from ThermoFisher Scientific.

Circular dichroism spectroscopy

Far-UV circular dichroism spectra of ID3 (0.1 mg/ml), ZFP10-f (0.1 mg/ml) and the complex in 1:1 ration were recorded on a J-715 spectropolarimeter in 50 mM KH_2PO4, 150 mM NaCl, 5 mM DTT buffer pH 6.5. Data points were collected every 0.5 nm at the controlled temperature of 24°C. Spectra are presented as the circular dichroism absorption coefficient calculated from the mean residue ellipticity (cf. Supplementary Fig. S1)[20].

Small-angle X-ray scattering experiments

The Aviex charge-coupled device detector was positioned at 1507.5 mm. A total volume of 60 µl of a ID3 sample (10 mg/ml) was injected into a size-exclusion column (SEC-3, 300 Ǻ Agilent), using an Agilent HPLC system, and eluted directly into the SAXS flow-through capillary cell at a flow rate of 0.2 ml $min^{-1}$and at 15°C[21]. The size-exclusion buffer used was 50 mM Tris, 150 mM NaCl, 5 mM Tcep, pH 6.5. SAXS data were collected continuously, with frame duration of 1.0 s and dead time of 0.5 s between frames. Selected frames corresponding to the main protein elution peak were averaged using FOXTROT[21]. A large number of frames were collected during the

void volume of the elution and averaged to account for buffer scattering, which was subsequently subtracted from the signals during the protein elution. Data reduction to absolute units, frame averaging and subtraction were done using FOXTROT. All subsequent data processing, analysis and modelling steps were carried out with the ATSAS suite[22]. The radius of gyration R_G was derived by the Guinier approximation $I(q) = I(0)\ \exp(-q^2R_G^2/3)$ for $qR_G < 1.0$ using PRIMUS QT[23]. GNOM was used to compute the pair-distance distribution functions, P(r)[24]. This approach also features the maximum dimension of the macromolecule, D_{max}. Normalized Kratky plots (i.e. $(qR_G)^2I(q)/I(0)$ as a function of qR_G) were used to assess the conformational behavior of the polypeptide chain[25].

Nuclear magnetic resonance (NMR) and paramagnetic relaxation enhancement (PRE) experiments

See also Supporting Information Tables S1-S5

1) Sequence-specific assignment:

All the multidimensional NMR experiments for sequence-specific assignment were performed on a sample of 0.4 mM uniformly [^{13}C,^{15}N] labeled ID3 in 50 mM KH_2PO_4, 150 mM NaCl, 2.5 mM TCEP buffer at pH 6.5, with 10% D_2O added for the lock.

A set of multidimensional $^1H^N$ and ^{13}C detected NMR experiments tailored to achieve sequence-specific assignment of IDPs was acquired at 297 K. 2D CON[26,27] 2D (HCA)CON[26,27], 2D BEST-CON[28], 3D (H)CACON[29], 4D HCBCACON[29], 4D HCBCANCO[29], 4D (HCA)CON(CA)CON[30], 4D ($H^{N\text{-flip}}$N)CON(CA)CON[30], 5D $H^{N\text{-flip}}$NCACON[29] and 5D $H^{N\text{-flip}}$NCANCO[29] experiments were acquired at 16.4 T on a Bruker Avance spectrometer operating at 700 MHz 1H, equipped with a cryogenically cooled probehead optimized for ^{13}C-direct detection. 2D BEST-TROSY (BT)[31,32], 3D TROSY-HNCO[31,33,34], 3D (H)N(CA)NNH[35], 4D TROSY-(H)NCO(CA)NNH[36] and 4D TROSY-HN(COCA)NNH[36] experiments were performed at 22.3 T on a Bruker Avance III spectrometer operating at 950 MHz, equipped with a cryogenically cooled probehead.

NMR experiments for sequence-specific assignment of ID3-F1 were acquired on a sample of 0.2 mM uniformly [^{13}C, ^{15}N] labeled protein in 50 mM KH_2PO_4, 150 mM NaCl, 5.0 mM TCEP buffer at pH 6.5, with 10% D_2O added for the lock. A series of multidimensional proton detected NMR experiments (2D BEST-TROSY, 3D BT-HNCO, 3D BT-HN(CA)CO[31,34], 3D BT-HNCACB[31,34], 3D BT-HN(CO)CACB[31,34] for sequence-specific assignment were acquired at 21.1 T on a Bruker Avance spectrometer operating at 900 MHz, equipped with a cryogenically cooled probehead.

The parameters used for the acquisition of all the experiments are reported in the Supporting information S1-S4 Tables. All the data sets were acquired using Bruker TopSpin 1.3 or 3.1 software. 3/4/5D experiments for the sequence-specific assignment were performed using on-grid non-uniform sampling (NUS). The on-grid ‘‘Poisson disk’’ sampling scheme [37] was chosen to generate the time schedules with the RSPack program (available at http://nmr.cent3.uw.edu.pl). The distribution was relaxation-optimized, i.e. the density of points was decaying according to the Gaussian distribution $\exp(-t^2/\sigma^2)$, with σ=0.5.

2) Backbone dynamics

Backbone dynamics (at 297 K) of 0.2 mM uniformly ^{15}N-labeled ID3 were studied by using ^{15}N relaxation data obtained from 2D ^1H-^{15}N HSQC-edited experiments[38]. ^{15}N spin-lattice relaxation rate constant (R_1), ^{15}N spin-spin relaxation rate constant (R_2) and steady-state heteronuclear ^1H -^{15}N NOEs were determined for about 85% of non-proline backbone amide ^{15}Ns. The experiments were performed in 50 mM KH_2PO_4, 150 mM NaCl, 2.5 mM TCEP buffer at pH 6.5, with 10% D_2O added for the lock. Heteronuclear ^{15}N relaxation experiments for the measurement of R_1, R_2 and ^{15}N-^1H NOEs were measured at 297 K at 16.4 T on a Bruker Avance spectrometer operating at 700 MHz, equipped with a cryogenically cooled probehead.

3) Paramagnetic relaxation enhancement (PRE) experiments

For the PRE measurements, 0.25 mM [^{13}C,^{15}N] ID3(C845S) and 1 molar equivalent (eq) of single-Cys ZFP106-f mutant tagged with EDTA(Mn^{2+}), were used for the paramagnetic samples. The diamagnetic references were done with wt ZFP106-f, as it has been previously shown that wt proteins can be considered equivalent to their EDTA(Zn^{2+}) tagged version for PRE referencing[39]. The control experiments were carried out on 0.25 mM [^{15}N,^{13}C] ID3(C845S) samples and 1 eq of wt hCSD1-EDTA(Mn^{2+}) (paramagnetic sample) or wt hCSD1 (diamagnetic sample). For the reduced-label control experiments, the same sample used for the I_{oxi}/I_{red} plots (at 0 and 150 mM) was desalted (HiTrap Desalting 5 mL, GE Healthcare) in 50 mM KH_2PO_4, 0.02% NaN_3, pH 6.5, concentrated and re-measured in absence and presence of reducing agents (addition of 2.5 mM DTT final concentration). All NMR samples contained 50 mM KH_2PO_4, 0.02 % NaN_3, pH 6.5 and 10 % D_2O for the lock. For the high salt measurements, NaCl was added to the sample to the final concentration of 150 mM. In case of wt references and control experiments (reduced label or wt hCSD1 diamagnetic reference), 2.5 mM DTT was added to the sample.

2D [^1H,^{15}N,] HSQC spectra of the paramagnetic and diamagnetic samples were acquired with 10 seconds repetition delay in order to determine I_{para}/I_{dia} intensity ratios as described elsewhere[39]. The parameters used for the acquisition of all the experiments are reported in the Supporting information S5 Tables.

For representation purpose of the I_{para}/I_{dia} plots a threshold value was calculated. To that aim the higher 10% values were left out and the following 20% were averaged. All the I_{para}/I_{dia} values were divided by this average to get the normalized PRE[40].

PRE experiments were performed at the Jean Jeener Center, VUB (Belgium) at 297k in a Varian Direct-Drive 800 MHz or 600 MHz NMR spectrometers, the latter equipped with a cold probe for enhanced sensitivity.

4) NMR data processing

Conventionally sampled NMR data sets were processed using Bruker *TopSpin 1.3* software, except those of PRE experiments, which were processed using *nmrPipe*[41]. NUS data sets were converted with *nmrPipe* and then processed using either the *Multidimensional Fourier Transform* (MFT) algorithm (for 3D data sets) or the *Sparse MFT* (SMFT) algorithm (for 4/5D data sets), implemented in *ToASTD*[42] and *reduced*[43,44] programs respectively. Both programs are available at http://nmr.cent3.uw.edu.pl.

Sparky [45] was used to analyze the spectra for sequence-specific assignment, whereas *CcpNmr Analysis*[46] was employed to analyze ^{15}N relaxation and PRE data.

Secondary structure propensity calculation

Secondary structure propensity analysis was performed using C', C^{α}, C^{β} and N chemical shifts (95% assigned). The ncSP from the heteronuclear chemical shifts was determined by using the *neighbor-corrected structural propensity calculator* (ncSP) tool[47], available online at http://nmr.chem.rug.nl. The random-coil chemical shift library derived from IDP NMR analysis was chosen for the analysis[48].

Ensemble modelling

Ensemble models of ID3 were based on the combination of SAXS data and ncSP derived from experimental chemical shift values. Flexible-Meccano[49] was used to generate a pool of 10.000 semi-random conformations; the conformational sampling was biased by ncSP. Side chains were modeled onto the backbone of the conformers by SCCOMP[50]. Then, the theoretical SAXS scattering curve of

each single conformer was calculated by CRYSOL[51], and finally the GAJOE algorithm was employed[51] to select subsets of the random pool that – when combined together – had a theoretical scattering curve that agreed with the experimental SAXS data.

Acetylation assay and identification of the acetylation sites

In vitro acetylation assays were performed in 50 µl buffer (50 mM NaH_2PO_4, 125 mM NaCl, 0.5 mM DTT, pH 7.4) at 30°C except for the time points of the H4 reaction by core domain- and full-length CBP that were done at 20°C (50mM Tris, 150 mM NaCl, 5% Glycerol, 50 µM Tcep, 0.02 mg/mL BSA, protease inhibitors, pH 7.5). The reactions were completed to saturation. For each reaction, 624 pmols of lyophilized ZFP106-f or histone 4 (H4, control reaction), 18.5 pmols of HAT-active full-length, and 20 µmols of acetyl-CoA were used. For competition experiments, ID3, scr-ID3 or ID3delCTR (control reactions) were added in 10-, 100- or 150-times molar excess to CBP. The samples were analyzed by WB by an anti-acetylated-lysine antibody (Bioke) and anti-His (Sigma-Aldrich).

For the identification of acetylation sites, the proteins in solution were desalted and concentrated on a C-18 spin column (Pierce, ThermoScientific) prior to an overnight pepsin or endoglu-C digest in 50 mM NH_4HCO_3 at 30°C. In case of SDS-PAGE gel band samples, the proteins were washed, completely dry in the vacuum system and digested with trypsin ON at 37°C in 50 mM NH_4HCO_3, pH 8.0, 10% ACN. The reactions were stopped by adding 0.1% trifluoroacetic acid. The peptides were analyzed by LC-MS/MS in a microelectrospray ionization ion trap mass spectrometer (LTQ XL, Thermo Scientific or Orbitrap XL) as described here [52,53]. For peptide identification, peak lists were generated using the application spectrum selector in the Proteome Discoverer 1.4 package. The resulting peak lists were searched using Sequest against a homemade protein database containing the protein sequences or the SwissProt *E. coli* database concatenated with full-length CBP and histone H4. Peptide matches were filtered using charge-state versus cross-correlation scores (Xcorr) and manually validated.

For the complete MS sequence coverage, two MS analyses were needed as each independent analysis covered only 62% and 90% of ZFP106-f amino acid sequence.

Cross-linking Assay

Cross-linking reactions were carried out for 15 or 30 min at RT in PBS containing 1 mM DTT (S: sample, ID3:ZFP106-f; C: control, GST:ZFP106-f). Proteins were mixed in approximate 1 to 1 ratio (concentrations adjusted by SDS-PAGE) and two different concentrations of the BS3 cross-linking reagent 0.25 mM and 2.5 mM were added to sample and control reaction. SDS-PAGE gels were run with samples before (12.5% SDS-PAGE gel; no cross-linker added) and after the cross-linking reaction (7,5% SDS-PAGE gel to improve the resolution of high Mw). The SDS-PAGE after the cross-linking was transferred to a blotting membrane and two western blots (anti-His and anti-GST with stripping step in between) were done in order to visualize the appearance of high Mw overlapping bands (formed complexes).

REFERENCES

1 Camacho, C. *et al.* BLAST+: architecture and applications. *BMC Bioinformatics* **10**, 421 (2009).

2 Katoh, K. & Standley, D. M. MAFFT multiple sequence alignment software version 7: improvements in performance and usability. *Mol Biol Evol* **30**, 772-780 (2013).

3 Varadi, M., Guharoy, M., Zsolyomi, F. & Tompa, P. DisCons: a novel tool to quantify and classify evolutionary conservation of intrinsic protein disorder. *BMC Bioinformatics* **16**, 153 (2015).

4 Dosztanyi, Z., Csizmok, V., Tompa, P. & Simon, I. The pairwise energy content estimated from amino acid composition discriminates between folded and intrinsically unstructured proteins. *J Mol Biol* **347**, 827-839 (2005).

5 Peng, K., Radivojac, P., Vucetic, S., Dunker, A. K. & Obradovic, Z. Length-dependent prediction of protein intrinsic disorder. *BMC Bioinformatics* **7**, 208 (2006).

6 McGuffin, L. J., Bryson, K. & Jones, D. T. The PSIPRED protein structure prediction server. *Bioinformatics* **16**, 404-405 (2000).

7 Dinkel, H. *et al.* The eukaryotic linear motif resource ELM: 10 years and counting. *Nucleic Acids Res* **42**, D259-266 (2014).

8 Disfani, F. M. *et al.* MoRFpred, a computational tool for sequence-based prediction and characterization of short disorder-to-order transitioning binding regions in proteins. *Bioinformatics* **28**, i75-83 (2012).

9 Dosztanyi, Z., Meszaros, B. & Simon, I. ANCHOR: web server for predicting protein binding regions in disordered proteins. *Bioinformatics* **25**, 2745-2746 (2009).

10 Cilia, E., Pancsa, R., Tompa, P., Lenaerts, T. & Vranken, W. F. From protein sequence to dynamics and disorder with DynaMine. *Nat Commun* **4**, 2741 (2013).

11 Walsh, I., Martin, A. J., Di Domenico, T. & Tosatto, S. C. ESpritz: accurate and fast prediction of protein disorder. *Bioinformatics* **28**, 503-509 (2012).

12 Rousseau, F., Schymkowitz, J. & Serrano, L. Protein aggregation and amyloidosis: confusion of the kinds? *Curr Opin Struct Biol* **16**, 118-126 (2006).

13 Tommaso, P. *et al.* T-Coffee: a web server for the multiple sequence alignment of protein and RNA sequences using structural information and homology extension. *Nucleic Acids Research* **39**, W13-W17 (2011).

14 McWilliam, H. *et al.* Analysis Tool Web Services from the EMBL-EBI. *Nucleic Acids Res* **41**, W597-600 (2013).

15 Dosztanyi, Z., Csizmok, V., Tompa, P. & Simon, I. IUPred: web server for the prediction of intrinsically unstructured regions of proteins based on estimated energy content. *Bioinformatics* **21**, 3433-3434 (2005).

16 UniProt, C. UniProt: a hub for protein information. *Nucleic Acids Res* **43**, D204-212 (2015).

17 Kraus, W. L. & Kadonaga, J. T. p300 and estrogen receptor cooperatively activate transcription via differential enhancement of initiation and reinitiation. *Genes Dev* **12**, 331-342 (1998).

18 Sivashanmugam, A. *et al.* Practical protocols for production of very high yields of recombinant proteins using Escherichia coli. *Protein Science* **18**, 936-948 (2009).

19 Livernois, A. M., Hnatchuk, D. J., Findlater, E. E. & Graether, S. P. Obtaining highly purified intrinsically disordered protein by boiling lysis and single step ion exchange. *Anal Biochem* **392**, 70-76 (2009).

20 Martin, S. R. & Schilstra, M. J. Circular dichroism and its application to the study of biomolecules. *Methods Cell Biol* **84**, 263-293 (2008).

21 David, G. & Perez, J. Combined sampler robot and high-performance liquid chromatography: a fully automated system for biological small-angle X-ray scattering experiments at the Synchrotron SOLEIL SWING beamline. *Journal of Applied Crystallography* **42**, 892-900 (2009).

22 Konarev, P. V., Petoukhov, M. V., Volkov, V. V. & Svergun, D. I. ATSAS 2.1, a program package for small-angle scattering data analysis. *Journal of Applied Crystallography* **39**, 277-286 (2006).

23 Konarev, P. V., Volkov, V. V., Sokolova, A. V., Koch, M. H. J. & Svergun, D. I. PRIMUS: a Windows PC-based system for small-angle scattering data analysis. *Journal of Applied Crystallography* **36**, 1277-1282 (2003).

24 Svergun, D. I. Determination of the Regularization Parameter in Indirect-Transform Methods Using Perceptual Criteria. *Journal of Applied Crystallography* **25**, 495-503 (1992).

25 Kratky, O. & Porod, G. Diffuse small-angle scattering of X-rays in colloid systems. *J Colloid Sci* **4**, 35-70 (1949).

26 Bermel, W. *et al.* Complete assignment of heteronuclear protein resonances by protonless NMR spectroscopy. *Angew Chem Int Ed Engl* **44**, 3089-3092 (2005).

27 Bermel, W., Bertini, I., Felli, I. C. & Pierattelli, R. Speeding up (13)C direct detection biomolecular NMR spectroscopy. *J Am Chem Soc* **131**, 15339-15345 (2009).

28 Gil, S. *et al.* NMR spectroscopic studies of intrinsically disordered proteins at near-physiological conditions. *Angew Chem Int Ed Engl* **52**, 11808-11812 (2013).

29 Bermel, W. *et al.* Speeding up sequence specific assignment of IDPs. *J Biomol Nmr* **53**, 293-301 (2012).

30 Bermel, W. *et al.* High-dimensionality 13C direct-detected NMR experiments for the automatic assignment of intrinsically disordered proteins. *J Biomol Nmr* **57**, 353-361 (2013).

31 Solyom, Z. *et al.* BEST-TROSY experiments for time-efficient sequential resonance assignment of large disordered proteins. *J Biomol Nmr* **55**, 311-321 (2013).

32 Pervushin, K., Vogeli, B. & Eletsky, A. Longitudinal (1)H relaxation optimization in TROSY NMR spectroscopy. *J Am Chem Soc* **124**, 12898-12902 (2002).

33 Salzmann, M., Pervushin, K., Wider, G., Senn, H. & Wuthrich, K. TROSY in triple-resonance experiments: new perspectives for sequential NMR assignment of large proteins. *Proc Natl Acad Sci U S A* **95**, 13585-13590 (1998).

34 Lescop, E., Schanda, P. & Brutscher, B. A set of BEST triple-resonance experiments for time-optimized protein resonance assignment. *J Magn Reson* **187**, 163-169 (2007).

35 Weisemann, R., Ruterjans, H. & Bermel, W. 3D triple-resonance NMR techniques for the sequential assignment of NH and 15N resonances in 15N- and 13C-labelled proteins. *J Biomol Nmr* **3**, 113-120 (1993).

36 Piai, A. *et al.* "CON-CON" assignment strategy for highly flexible intrinsically disordered proteins. *J Biomol Nmr* **60**, 209-218 (2014).
37 Kazimierczuk, K., Zawadzka, A. & Kozminski, W. Optimization of random time domain sampling in multidimensional NMR. *J Magn Reson* **192**, 123-130 (2008).
38 Barbato, G., Ikura, M., Kay, L. E., Pastor, R. W. & Bax, A. Backbone dynamics of calmodulin studied by 15N relaxation using inverse detected two-dimensional NMR spectroscopy: the central helix is flexible. *Biochemistry* **31**, 5269-5278 (1992).
39 Van de Water, K., van Nuland, N. A. J. & Volkov, A. N. Transient protein encounters characterized by paramagnetic NMR. *Chem Sci* **5**, 4227-4236 (2014).
40 Bashir, Q., Volkov, A. N., Ullmann, G. M. & Ubbink, M. Visualization of the encounter ensemble of the transient electron transfer complex of cytochrome c and cytochrome c peroxidase. *J Am Chem Soc* **132**, 241-247 (2010).
41 Delaglio, F. *et al.* Nmrpipe - a Multidimensional Spectral Processing System Based on Unix Pipes. *J Biomol Nmr* **6**, 277-293 (1995).
42 Kazimierczuk, K., Zawadzka, A., Kozminski, W. & Zhukov, I. Random sampling of evolution time space and Fourier transform processing. *J Biomol Nmr* **36**, 157-168 (2006).
43 Kazimierczuk, K., Zawadzka, A. & Kozminski, W. Narrow peaks and high dimensionalities: exploiting the advantages of random sampling. *J Magn Reson* **197**, 219-228 (2009).
44 Kazimierczuk, K., Zawadzka-Kazimierczuk, A. & Kozminski, W. Non-uniform frequency domain for optimal exploitation of non-uniform sampling. *J Magn Reson* **205**, 286-292 (2010).
45 Goddard, T. D. *Sparky - NMR Assignment and Integration Software*
46 Vranken, W. F. *et al.* The CCPN data model for NMR spectroscopy: Development of a software pipeline. *Proteins* **59**, 687-696 (2005).
47 Tamiola, K. & Mulder, F. A. Using NMR chemical shifts to calculate the propensity for structural order and disorder in proteins. *Biochem Soc Trans* **40**, 1014-1020 (2012).
48 Tamiola, K., Acar, B. & Mulder, F. A. A. Sequence-Specific Random Coil Chemical Shifts of Intrinsically Disordered Proteins. *Journal of the American Chemical Society* **132**, 18000-18003 (2010).
49 Ozenne, V. *et al.* Flexible-meccano: a tool for the generation of explicit ensemble descriptions of intrinsically disordered proteins and their associated experimental observables. *Bioinformatics* **28**, 1463-1470 (2012).
50 Eyal, E., Najmanovich, R., McConkey, B. J., Edelman, M. & Sobolev, V. Importance of solvent accessibility and contact surfaces in modeling side-chain conformations in proteins. *J Comput Chem* **25**, 712-724 (2004).
51 Petoukhov, M. V. *et al.* New developments in the program package for small-angle scattering data analysis. *J Appl Crystallogr* **45**, 342-350 (2012).
52 Ruys, S. P. D. *et al.* Identification of autophosphorylation sites in eukaryotic elongation factor-2 kinase (vol 442, pg 681, 2012). *Biochem J* **443**, 328-328 (2012).
53 Helsens, K. *et al.* ms_lims, a simple yet powerful open source laboratory information management system for MS-driven proteomics. *Proteomics* **10**, 1261-1264 (2010).
54 Chevillard-Briet, M., Trouche, D. & Vandel, L. Control of CBP co-activating activity by arginine methylation. *EMBO J* **21**, 5457-5466 (2002).
55 Weinert, B. T. *et al.* Lysine succinylation is a frequently occurring modification in prokaryotes and eukaryotes and extensively overlaps with acetylation. *Cell Rep* **4**, 842-851 (2013).
56 Kuo, H. Y. *et al.* SUMO modification negatively modulates the transcriptional activity of CREB-binding protein via the recruitment of Daxx. *Proc Natl Acad Sci U S A* **102**, 16973-16978 (2005).
57 Matsuoka, S. *et al.* ATM and ATR substrate analysis reveals extensive protein networks responsive to DNA damage. *Science* **316**, 1160-1166 (2007).

SUPPLEMENTARY FIGURES AND FIGURE LEGENDS

Fig. S1. 2D HN and CON spectra of ID3 and quality-check on the heat-treatment step during ID3 purification. (**a**) Comparison of 2D HN and CON spectra of ID3 highlights the improvement on the signal dispersion obtained by applying ^{13}C-direct detection methods. (**b**) Circular dichroism intensities of ID3 non-boiled (light green) and ID3 boiled (dark green) given as a mean residue ellipticity ($[\theta]_{mrw}$). (**c**) The superimposition of the 2D BEST-TROSY spectra of boiled (black) and non-boiled (red) ID3 sample.

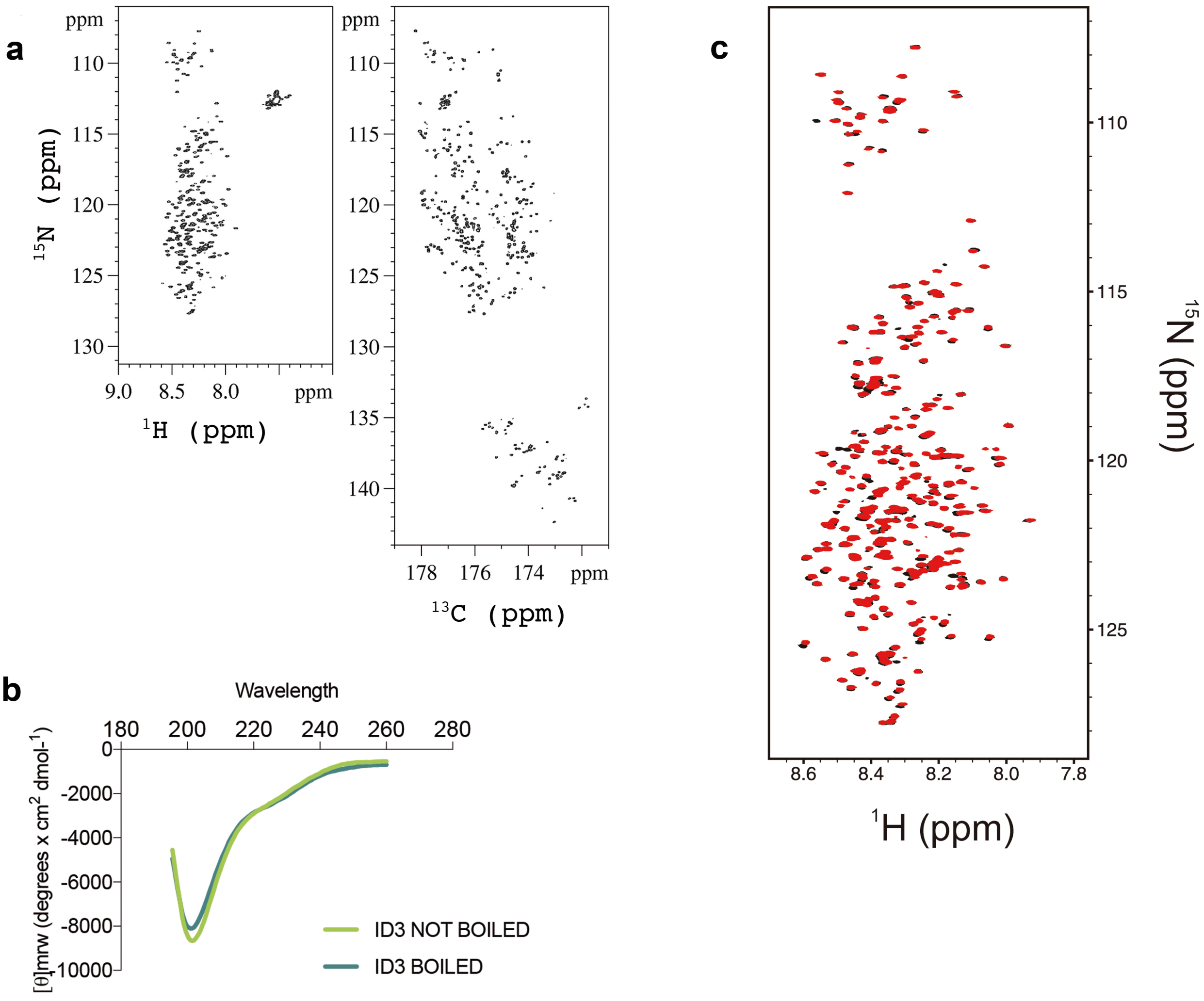

Fig. S2. Structural and dynamic characterization of ID3 by NMR. Dynamic characterization of ID3: (**a**) ^{15}N R_1 relaxation rates; (**b**) ^{15}N R_2 relaxation rates; (**c**) ^{15}N-^{1}H NOEs.

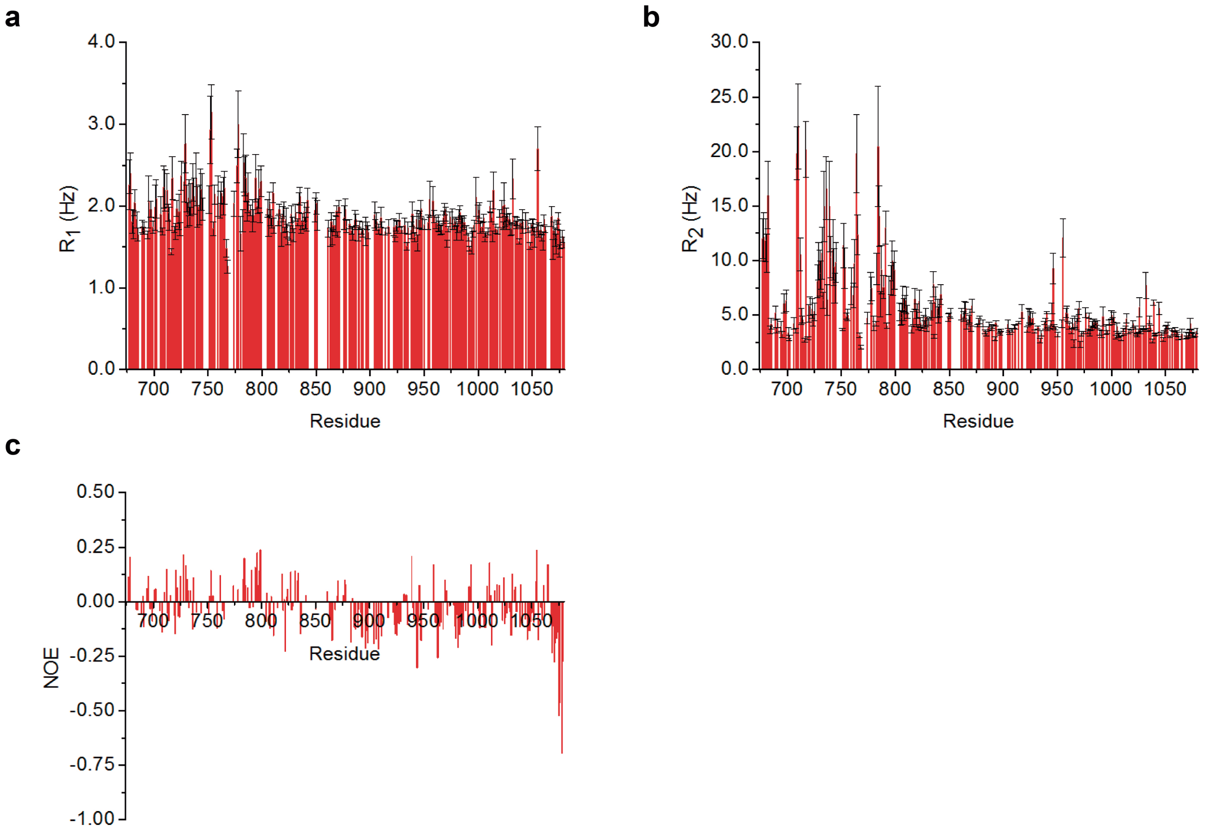

Fig. S3. Ensemble fit and ID3 preference for binding disordered regions of the identified proteins. (**a**) Experimental scattering curve of the SAXS measurements (green) and the fit of the selected ensemble (teal). (**b**) Density plot. Paired Wilcoxon Mann-Whitney statistical test gives a p-value of 0.03808, indicating statistically significant difference between the distributions of the ratios of disordered residues in fragments (red) versus their full-length proteins (black).

a

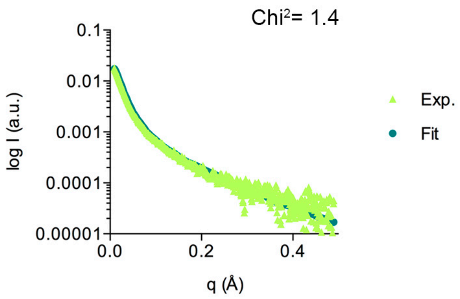

b

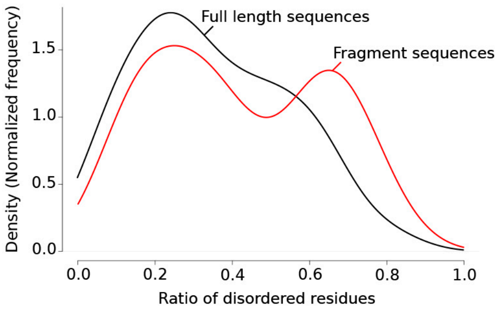

Fig. S4. Observed and predicted sequence features of ID3. Experimental and in silico analyses of sequence features of potential functional importance. The ncSP scores plotted on the bottom show regions with a tendency to form helices (y > 0) or sheet (y < 0). Missing segments correlates to unassigned residues. This (experimental) information correlates with backbone dynamics predicted with DynaMine and predicted MoRFs. Interestingly, the more pronounced helices derived from ncSP scores correspond to DynaMine scores associated with context dependence. DynaMine profile peaks observed at the C-terminal region of ID3 (underlined). There are several potential ANCHOR binding regions scattered along the sequence, and sequence conservation also suggests the functional importance of a significant portion of ID3. Some highly conserved segments also correspond to predicted MoRFpred sites. One segment (~824-844) is also observed as a transient helix in the ensemble model of ID3. A high density of post-translational modification (PTM) sites points to the functional importance of the C-terminal region of ID3 (*Methylation*[54] *in red; Acetylation*[55] *in blue; SUMOylation*[56] *in orange; Phosphorylation*[57] *in purple*.

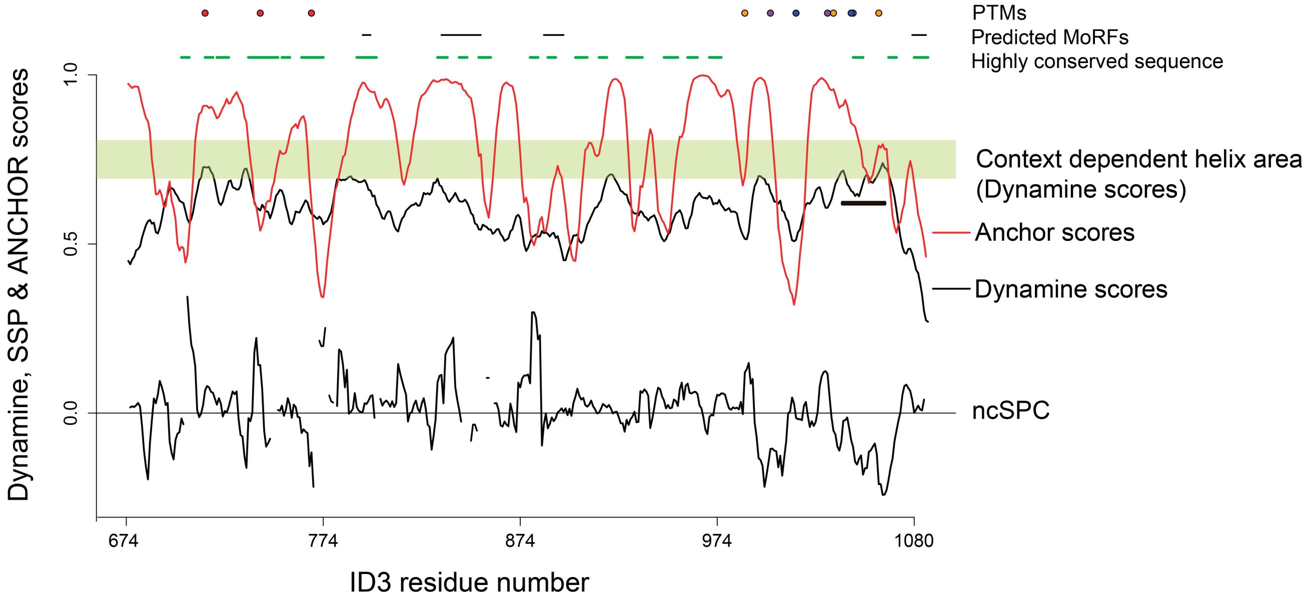

Fig. S5. ID3–ZFP106-f interaction assessed by competition binding assay. (**a**) Competition assays demonstrate that recombinant ID3 competes with ID3 in the full-length CBP for binding ZFP106-f, which is shown by a concentration-dependent decrease on the ZFP106-f acetylation in presence of an excess of recombinant ID3 (WB developed with anti-acetylated-lysine antibody). In contrast, addition of the same amount of scrambled ID3 (scrID3) did not cause any effect (control experiment 1). (**b**) Control experiment 2: The same experiments were run using histone 4 as a substrate, and no changes on H4 acetylation level could be detected after addition of an excess of ID3. Acetylation was followed by anti-Ac-Lys antibody, ID3 was added to the acetylation reaction in an excess of 10, 100 or 150 times to full-length CBP (18.5 pmol) and its effect was also compared to that of scrambled-ID3 (scrID3; control reaction) *(**C+**: control reaction)*. Loading controls are shown with the SDS-PAGE and WB developed with anti-His antibody (both ID3 and ZFP106-f are his-tagged). (**c**) Competition assays demonstrate that recombinant ID3 competes with ID3 in the full-length CBP for binding ZFP106-f, which is shown by a concentration-dependent decrease on the ZFP106-f acetylation in presence of an excess of recombinant ID3 (WB developed with anti-acetylated-lysine antibody). In contrast, addition of the same amount of a C-terminal deletion mutant of ID3 (ID3delCTR) did not cause any effect (control experiment 3). Note: The apparent Mw on SDS-PAGE gel of ID3delCTR and ZFP106-f is the same. Therefore, addition of an excess of ID3delCTR is seen as an increase in thickness of ZFP106-f band (or just above it), without this perturbing the WB analysis of acetylation.

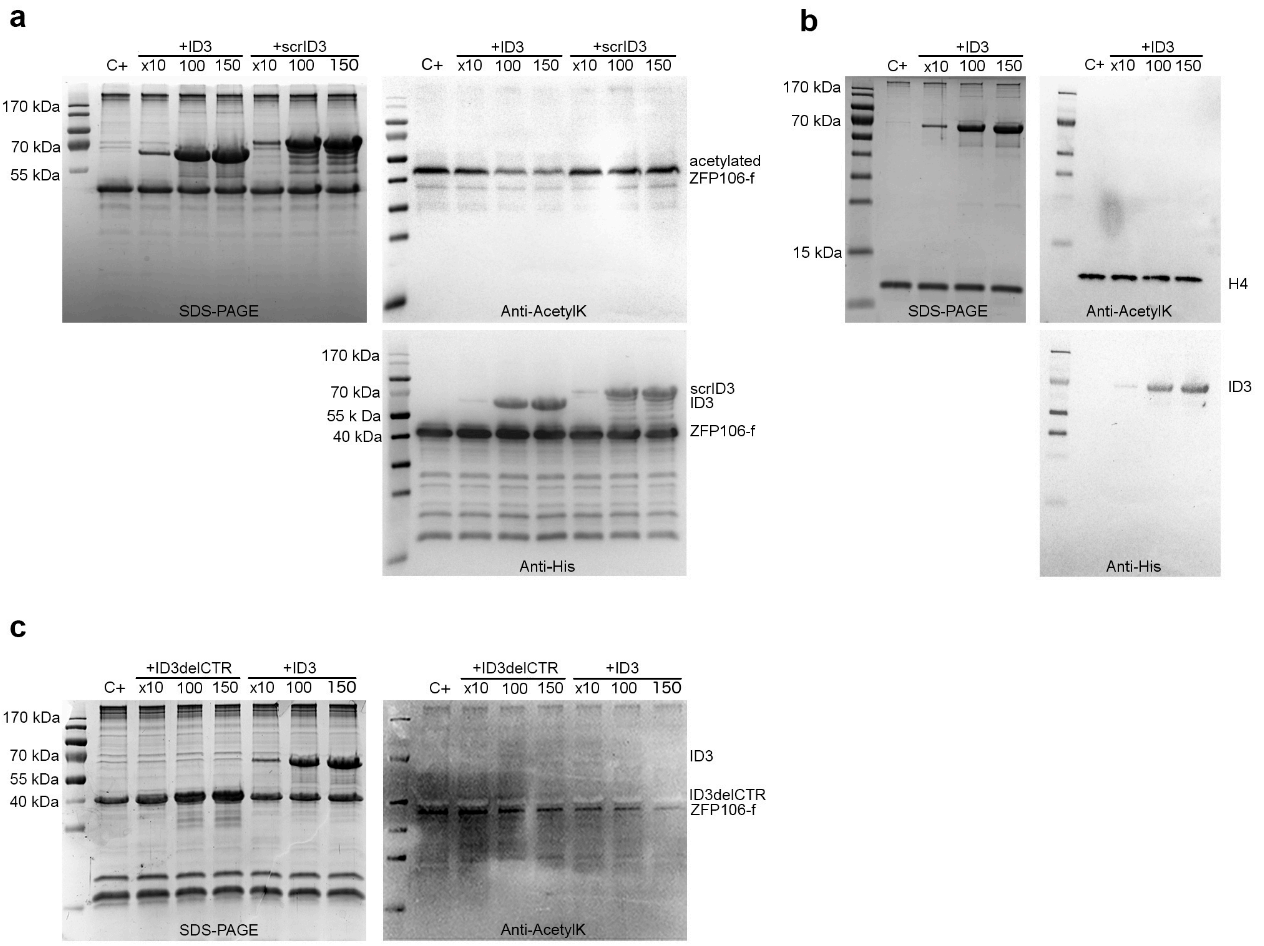

Fig. S6. ID3 and ZFP106-f can be specifically cross-linked "in vitro"

(**a**) In the *SDS-PAGE gel before* the proteins of sample (*S*: sample containing ID3 - at 100 kDa - and ZFP106-f - about 40 kDa-) and control (C: control containing GST - 25 kDa – and ZFP106-f- about 40 kDa -) previous to the cross-linking reaction are shown. (**b**) Cross-linking reactions were carried out for 15 or 30 min at RT and, two different concentrations of the BS3 cross-linker (0.25 mM and 2.5 mM) were added to sample and control reaction (Note: Excess of the cross-linker reagent results in the nonspecific cross-linking of all species present in the reaction). *SDS-PAGE gels after* the cross-linking reaction, together with two western blots (anti-His and anti-GST with stripping step in between) allow us to visualize the appearance of high Mw overlapping bands corresponding to ZFP106-f – ID3 complex (red box). Specificity of the reaction was assessed by the control reaction, where under similar conditions, a complex between ZFP106-f and GST was not observed. However, we could identify the formation of other complexes such as ZFP106-f and GST dimer.

a

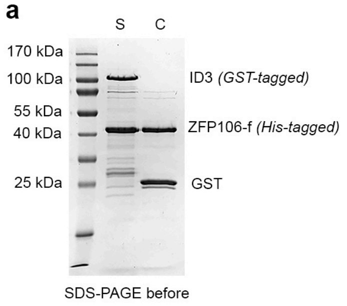

b

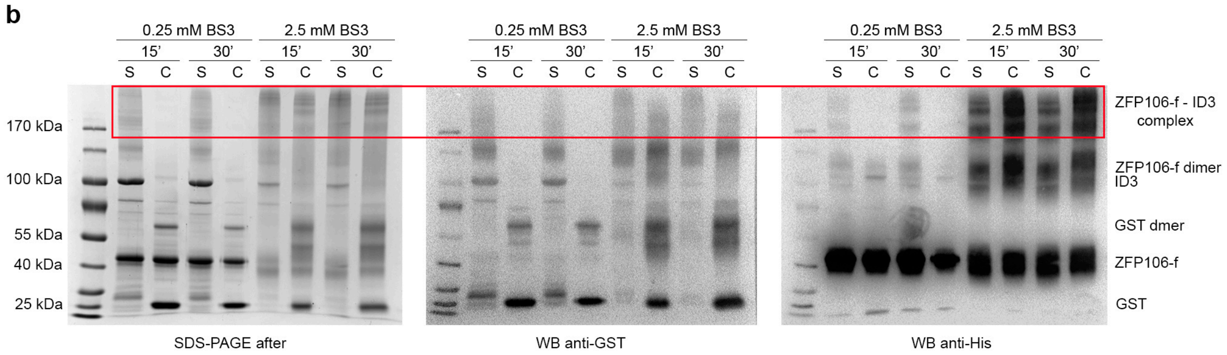

Fig. S7. C-terminal region (CTR) of ID3 interacts with the middle region of ZFP106-f. (**a**) NMR titration of ID3 and ZFP106-f. Garrett plot of ID3 chemical shift perturbations upon adding 7.8 equivalents of ZFP106-f. (**b** – **e**) I_{para}/I_{dia} plots of the HSQC cross-peaks of ID3 in the presence of the paramagnetically tagged single-Cys ZFP106-f mutants M1, M2 and M3 or hCSD1 (control) under different conditions. A non-specific interaction between the label attached to the protein and the NTR of ID3 was observed in all experiments (blue). In contrast, only ZFP106-f-M2 caused PRE effect at the CTR of ID3 (red) due to the specific interaction between the two protein fragments. This signal diminished in the presence salt as shown by I_{para}/I_{dia} values in the absence (**b**) and presence (**c**) of 150 mM NaCl. Control experiments (**d**) reduced label and (**e**) unrelated protein were carried out only at 0 mM NaCl.

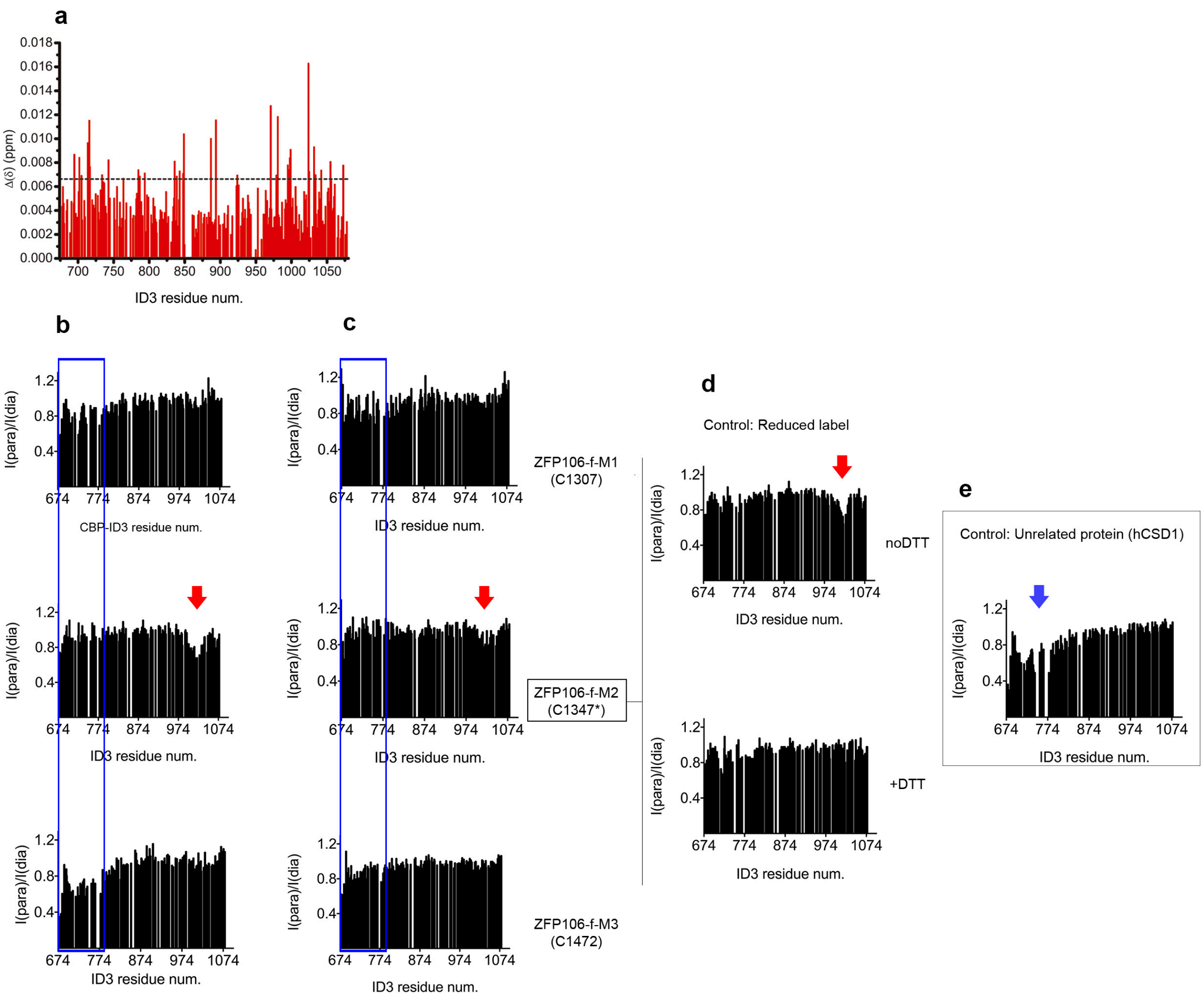

Fig. S8. Scrambled-ID3 sequence and alignment with human ID3. Scrambled ID3 (scrID3) sequence generated as control for the acetylation competition assay. Identical or similar residues in the multiple-alignment chart are represented by black or grey, respectively. ID3 and scrID3 sequence alignment shows a level of amino acid identity lower than 30%.

>scrID3

QPSSGATPQGAEVTPPMVEEDQQAAGQPGSRVGQTSHAHSLPPSSGNSNAPEPISTAPPNTKVPMQPATSVPAQQMRMPSTLKQQPNMSSEQTATIMMPSDAIV
ANGQPSRTSPHQLNLSARGTQANKQVQVPRKLPGVPQLPCQPNKAPKMGPAEPSGLSANSQPSEVASPVQQASVSEKPNPVMQEGTQASVAPAPTNQSPSPNAL
QGPPPPQGAPMAPSMVGSPTAVSPAATQSFDIPVGSEMGGADDSSQPTPPAPQATTDQGTPSGEREPDNLGEETMQQHQTVPGLTPPQVPPQATAAMPMQQKQ
LPPPSAPPMVFNPSMGELAQLGPSEQPQGQTVPSPPTSHPNESPTFEEQTQAPQMMSTNLTQSGPEASVTGIQQVPEMVSMHVVSPPELKPMNSLV

```
ID3        1 HKQGILGNQPALPAPGAQPPVIPQAQPVRPPN-----GPLSLPVNRMQVSQGMNSFNPMS
scrID3     1 Q-----------PSSGAT----PQGAEVTPPMVEEDQQAAGQPGSRVGQTSHAHSLPP-S

ID3       56 LGNVQL QAPMGPRAASPMNHSVQMNSMGSVPGMAI-SPSRMPQPPNMMGAHTNNMMAQA
scrID3    45 SGNSNAPE----PISTAPPNTKVPMQPATSVPAQQMRMPSTLKQQPNMSSEQTATIMMPS

ID3      115 PA-------------------------------QSQFLPQ-----NQFPSSSGAMSVGM
scrID3   101 DAIVANGQPSRTSPHQLNLSARGTQANKQVQVPRKLPGVPQLPCQPNKAPKMGPAEPSGL

ID3      138 --GQPPAQTGVSQGQVPGAALPNPLNMLGPQASQLPCPPVTQSPLH---PTPPPASTAAG
scrID3   161 SANSQPSEVASPVQQASVSEKPNPVMQEGTQASVAPAP-TNQSPSPNALQGPPPPQGAPM

ID3      193 MPSLQHTTPPGMTPPQPAAPTQPSTPVSS----SGQTPTPTPGSVPSATQTQSTPTVQA-
scrID3   220 APSMVG-SPTA---VSPAATQSFDIPVGSEMGGADDSSQPTPP-APQATTDQGTPSGERE

ID3      248 --------AAQAQVTPQPQTPVQPPSVATPQSSQQQPTPVHAQPPGTP------LSQAAA
scrID3   275 PDNLGEETMQQHQTVPGLTPPQVPPQATAAMPMQQKQLPPPSAPPMVFNPSMGELAQLG-

ID3      294 SIDNRVPTPSSVASAETNSQQPGPDVPVLEMKTETQAEDTEPDPGESKGEPRS-------
scrID3   334 --PSEQPQ----------------------------GQTVPSPPTSHPN-ESPTFEEQT

ID3      347 ---EMM---------EEDLQGASQVKEETDIAEQKSEPMEVDEKKPEVKVEVKEEEESSS
scrID3   362 QAPQMMSTNLTQSGPEASVTGIQQVPEMVS--MHVVSPPELKPMNSLV------------

ID3      395 NGTASQSTSPSQP
scrID3       -------------
```

SUPPLEMENTARY TABLES

Table S1 Experimental parameters used for the acquisition of the NMR experiments on ID3 for sequence-specific assignment.

	Spectral widths and maximal evolution times					No. of scans	Inter-scan delay (s)	No. of complex points (aq)	No. of hypercomplex points	Duration of the experiment	Relative data points density (%)
2D BEST-TROSY	2200 Hz (^{15}N) 231.2 ms	11400 Hz (^{1}H^{N}) 358.3 ms				4	0.005	4096	512	40 min	100.0
2D CON-IPAP	2700 Hz (^{15}N) 94.8 ms	8800 Hz (^{13}C') 58.3 ms				16	2.5	512	256	12 h 30 min	100.0
2D (HACA)CON-IPAP	2700 Hz (^{15}N) 94.8 ms	8800 Hz (^{13}C') 58.3 ms				16	1.2	512	256	6 h 50 min	100.0
2D BEST-CON-IPAP	1600 Hz (^{15}N) 31.3 ms	5300 Hz (^{13}C') 96.8 ms				64	0.4	512	50	3 h	100.0
3D (H)CACON-IPAP	4800 Hz (^{13}C$^{\alpha}$) 25.6 ms	2700 Hz (^{15}N) 40.0 ms	8800 Hz (^{13}C') 58.3 ms			8	1.2	512	1250	1 d 7 h 25 min	9.3
3D HN(CA)NNH	2000 Hz (^{15}N) 23.5 ms	2000 Hz (^{15}N) 21.5 ms	9800 Hz (^{1}H^{N}) 105.0 ms			32	1.0	1024	770	1 d 11 h	36.5
3D TROSY HNCO	2300 Hz (^{13}C') 30.4 ms	2300 Hz (^{15}N) 21.8 ms	14300 Hz (^{1}H^{N}) 71.9 ms			8	1.2	1024	685	8 h 30 min	20.0
4D (H)CBCACON-IPAP	3400 Hz (^{1}H$^{\alpha/\beta}$) 10.0 ms	11200 Hz (^{13}C$^{\alpha/\beta}$) 6.8 ms	2700 Hz (^{15}N) 30.0 ms	8800 Hz (^{13}C') 58.3 ms		8	1.1	512	1085	2 d 3 h 40 min	48.8
4D (H)CBCANCO-IPAP	3400 Hz (^{1}H$^{\alpha/\beta}$) 10.0 ms	11200 Hz (^{13}C$^{\alpha/\beta}$) 6.8 ms	2700 Hz (^{15}N) 31.9 ms	8800 Hz (^{13}C') 58.3 ms		16	1.2	512	775	3 d 12 h	32.8
4D (HACA)CON(CA)CON-IPAP	2000 Hz (^{13}C') 28.3 ms	3000 Hz (^{15}N) 24.3 ms	3000 Hz (^{15}N) 24.0 ms	8800 Hz (^{13}C') 58.3 ms		16	1.2	512	1030	4 d 16 h 20 min	0.33
4D (H$^{N\text{-}flip}$N)CON(CA)CON-IPAP	2000 Hz (^{13}C') 22.0 ms	3000 Hz (^{15}N) 23.0 ms	3000 Hz (^{15}N) 26.0 ms	8800 Hz (^{13}C') 58.3 ms		32	0.7	512	950	5 d 18 h 30 min	0.36
4D TROSY (H)NCO(CA)NNH	2300 Hz (^{15}N) 26.1 ms	2300 Hz (^{13}C') 26.1 ms	2300 Hz (^{15}N) 26.7 ms	13300 Hz (^{1}H^{N}) 77.0 ms		8	1.2	1024	2900	3 d 15 h	1.4
4D TROSY HN(COCA)NNH	1100 Hz (^{1}H^{N}) 24.5 ms	2300 Hz (^{15}N) 26.1 ms	2300 Hz (^{15}N) 26.7 ms	13300 Hz (^{1}H^{N}) 77.0 ms		8	1.2	1024	1360	1 d 11 h	1.4
5D H$^{N\text{-}flip}$NCACON-IPAP	1000 Hz (^{1}H^{N}) 10.0 ms	1700 Hz (^{15}N) 27.6 ms	4800 Hz (^{13}C$^{\alpha}$) 27.3 ms	2700 Hz (^{15}N) 22.3 ms	8800 Hz (^{13}C') 58.3 ms	16	0.5	512	620	2 d 23 h	0.016
5D H$^{N\text{-}flip}$NCANCO-IPAP	1000 Hz (^{1}H^{N}) 10.0 ms	1700 Hz (^{15}N) 20.6 ms	4800 Hz (^{13}C$^{\alpha}$) 41.7 ms	2700 Hz (^{15}N) 29.3 ms	8800 Hz (^{13}C') 116.4 ms	32	0.5	1024	575	6 d 1 h	0.009

Table S2 Experimental parameters used for the acquisition of ^{15}N relaxation NMR experiments.

	Spectral widths and maximal evolution times		No. of scans	Inter-scan delay (s)	No. of complex points (aq)	Duration of the experiment
^{15}N R_1	1700 Hz (^{15}N) 150.3 ms	10500 Hz (^{1}H^{N}) 97.6 ms	8	3.0	256	3 h 45 min – 4 h 50 min
^{15}N R_2	1700 Hz (^{15}N) 150.3 ms	10500 Hz (^{1}H^{N}) 97.6 ms	8	3.0	256	4 h 10 min
Steady-state heteronuclear ^{15}N{^{1}H} NOEs	1700 Hz (^{15}N) 150.3 ms	10500 Hz (^{1}H^{N}) 97.6 ms	80	6.0	256	3 d 5 h
For the determination of R_1, 10 experiments were acquired changing the variable delay from 15.0 ms to 1.0 s.<br>For the determination of R_2, 10 experiments were acquired changing the variable delay from 30.0 to 410.0 ms.						

Table S3 Experimental parameters used for the NMR titration between ID3 and ZFP106.

	Spectral widths and maximal evolution times		No. of scans	Inter-scan delay (s)	No. of complex points (aq)	Duration of the experiment
2D BEST-TROSY	2200 Hz (^{15}N) 231.2 ms	11400 Hz (^{1}H^{N}) 358.3 ms	16	0.005	4096	2 h 30 min

Table S4 Experimental parameters used for the acquisition of the NMR experiments on ID3-F1 for sequence-specific assignment.

	Spectral widths and maximal evolution times			No. of scans	Inter-scan delay (s)	No. of complex points (aq)	No. of hypercomplex points	Duration of the experiment	Relative data points density (%)
2D BEST-TROSY	2000 Hz (^{15}N) 256.0 ms		10800 Hz (^{1}H^{N}) 95.1 ms	4	0.2	1024	512	35 min	100.0
3D BT-HNCO	2500 Hz (^{13}C') 59.6 ms	2000 Hz (^{15}N) 63.5 ms	10800 Hz (^{1}H^{N}) 95.1 ms	4	0.2	1024	2900	5 h 35 min	15.0
3D BT-HN(CA)CO	2500 Hz (^{13}C') 59.6 ms	2000 Hz (^{15}N) 63.5 ms	10800 Hz (^{1}H^{N}) 95.1 ms	4	0.2	1024	2900	5 h 40 min	15.0
3D BT-HNCACB	16000 Hz (^{13}C$^{\alpha/\beta}$) 21.8 ms	2000 Hz (^{15}N) 63.5 ms	10800 Hz (^{1}H^{N}) 95.1 ms	4	0.2	1024	3700	7 h 20 min	8.5
3D BT-HN(CO)CACB	2000 Hz (^{15}N) 28.5 ms	2000 Hz (^{15}N) 63.5 ms	10800 Hz (^{1}H^{N}) 95.1 ms	32	0.2	1024	3300	2d 5 h 45 min	44.0

Table S5 Experimental parameters used for the PRE NMR experiments between ID3 and ZFP106-f-M1/2/3 or hCSD1.

	Spectral widths and maximal evolution times		No. of scans	Inter-scan delay (s)	No. of complex points (aq)	Duration of the experiment
[^{1}H,^{15}N] HSQC	1300 Hz (^{15}N) 100.3 ms	9800 Hz (^{1}H^{N}) 104.8 ms	16	10.0	1024	11 h 40 min
